# An Unorthodox Enolate–Triggered Radical Relay Directs the Chemo Upgrading of Levulinic Acid Into Citramalic Acid

**DOI:** 10.1002/cssc.202502757

**Published:** 2026-02-24

**Authors:** Geun Ho Kim, Young Kwang Han, Tae Woo Lee, Eun Jeong Yoo, Jung Woon Yang

**Affiliations:** ^1^ Department of Energy Science Sungkyunkwan University Suwon Republic of Korea; ^2^ Department of Applied Chemistry Kyung Hee University Yongin Republic of Korea

**Keywords:** biofuel precursor, biomass valorization, citramalic acid, sustainable chemistry, transition‐metal‐free oxidation

## Abstract

We report a transition‐metal‐free and highly selective chemical transformation of levulinic acid into citramalic acid via *t*‐butoxide‐mediated enolate chemistry, complementing both the biotransformation of glucose and glycerol using *E. coli* as well as transition‐metal‐catalyzed processes based on levulinic acid. The atypical behavior of *t*‐butoxide—classically recognized as a base that favors kinetic enolate formation in carbonyl chemistry—proves crucial for accessing the thermodynamic enolate under elevated temperatures and extended reaction times, thereby directing the reaction toward citramalic acid as the major product. Radical‐trapping experiments with TEMPO (2,2,6,6‐tetramethylpiperidine 1‐oxyl) and benzoic acid significantly diminished the product yield, indicating the involvement of radical intermediates in the initial oxygenation sequence and providing clear mechanistic insight into the operative pathway. Moreover, citramalic acid serves as a sustainable, bio‐based platform chemical that is amenable to downstream valorization into high‐value feedstocks, such as unnatural amino acid derivative and itaconic acid.

## Graphical abstract

**Unorthodox Thermodynamic** **Enolate Coupled with a Radical Pathway** A transition‐metal‐free oxidation of biomass‐derived levulinic acid to citramalic acid is achieved through an unorthodox enolate in combination with a radical pathway using potassium *tert*‐butoxide (KO*t*Bu) and molecular oxygen (O_2_). The transformation is readily scalable and provides citramalic acid as a versatile platform for accessing propene precursors, unnatural amino acid derivatives, and itaconic acid.

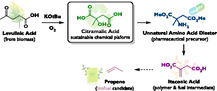

## 1 Introduction

The global shift toward a low‐carbon and sustainable economy has intensified interest in bio‐based platform chemicals as renewable alternatives to their fossil‐derived counterparts [1, 2, 3, 4, 5]. Among these, citramalic acid (2‐hydroxy‐2‐methylsuccinic acid) has emerged as a particularly attractive candidate owing to its structural versatility and broad potential applications in the production of biodegradable materials, environmentally benign solvents, and high‐value chemical intermediates [6, 7, 8]. As a C5 *α*‐hydroxy dicarboxylic acid structurally analogous to malic acid, citramalic acid holds significant promise as a precursor for the development of eco‐friendly industrial processes [9, 10, 11].

**SCHEME 1:**
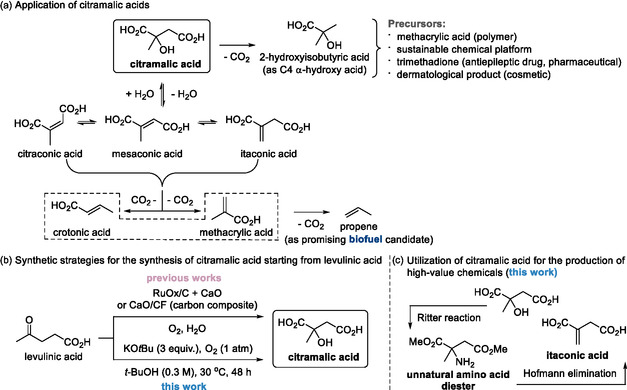
a) Diverse applications of citramalic acid in bio‐based chemical platforms and biofuels b) previous and current synthetic strategies for citramalic acid c) current sustainable valorization techniques for the conversion of citramalic acid into valuable feedstocks.

To date, citramalic acid has been produced primarily via microbial fermentation using engineered strains such as *Escherichia coli* (*E. coli*), in which renewable carbon sources are converted through the action of citramalate synthase (CimA) [12, 13, 14, 15]. Although these biotechnological routes are often regarded as inherently sustainable, their practical implementation at scale remains challenging. In particular, the heterologous expression of CimA introduces metabolic bottlenecks and product‐associated cellular stress that limit achievable titers, while the extended cultivation times required for fermentation result in low space–time yields and a substantial operational footprint. Moreover, the isolation of highly water‐soluble organic acids from diluted aqueous broths necessitates multistep, energy‐intensive downstream processing, including acidification, extraction, and purification, thereby eroding the overall sustainability and economic viability of the process [14, 15]. As a result, these constraints severely impede industrial productivity and scalability relative to chemical manufacturing routes. Given the broad applicability of citramalic acid in polymers, pharmaceuticals, cosmetics, and biofuels (Scheme 1a) [7], alternative chemical strategies starting from biomass‐derived levulinic acid have been explored.

Whereas the reductive upgrading of levulinic acid to biofuel precursors via catalytic transfer hydrogenation has been extensively investigated [16, 17], oxidative pathways to functionalized derivatives remain comparatively underexplored. Thus far, however, only two chemical approaches have been reported for the conversion of biomass‐derived levulinic acid: aerobic transition‐metal‐catalyzed oxidations and carbon‐based composite‐mediated reactions conducted in the presence of calcium oxide (CaO) (Scheme 1b, top) [18, 19]. Although these methods demonstrate proof‐of‐concept feasibility, they rely heavily on heterogeneous catalysts or engineered composite materials that require nontrivial synthesis, pretreatment, and regeneration. In addition, the need for elevated temperatures and pressurized oxygen environments substantially increases energy input and process complexity. Most critically, the involvement of transition metals raises persistent concerns regarding residual metal contamination, catalyst leaching, and long‐term stability, which constitute major regulatory and practical obstacles for applications targeting biocompatible polymers, pharmaceuticals, or food‐grade additives.

Therefore, the development of a practical chemical process that reconciles sustainability with operational simplicity and industrial productivity remains highly desirable. Herein, we report an unexpectedly efficient, transition‐metal‐free, base‐mediated oxidation of levulinic acid to citramalic acid that proceeds under ambient conditions using molecular oxygen as the sole oxidant (Scheme 1b, bottom). In sharp contrast to existing chemical approaches that depend on prefabricated heterogeneous catalysts, elevated temperatures, or pressurized oxygen, the present KO*t*Bu–O_2_ system operates without transition metals, specialized catalyst synthesis, or energy‐intensive conditions. By employing molecular oxygen as a ubiquitous, inexpensive, and atom‐economical oxidant in combination with a commercially available alkoxide base, this protocol avoids issues related to metal contamination, catalyst deactivation, and regulatory compliance, thereby providing a sustainable and scalable solution that overcomes the intrinsic limitations of both fermentation‐based and metal‐catalyzed routes. Mechanistic studies reveal that levulinic acid is funneled through an unorthodox thermodynamic enolate [20, 21, 22, 23, 24], triggering a radical relay pathway that enables the chemo‐upgrading of levulinic acid to citramalic acid under a molecular oxygen atmosphere [25]. The pivotal oxygenation event is cooperatively governed by enolate equilibration, as substantiated by deuterium‐labeling experiments, and by the intervention of bona fide radical intermediates, as evidenced by radical‐trapping studies. Beyond the core transformation, the reaction platform exhibits excellent scalability and enables direct downstream molecular diversification, including the synthesis of an unnatural amino acid derivative and itaconic acid (Scheme 1c), thereby demonstrating its value as a sustainable and versatile platform for biomass upgrading and industrially relevant chemical production.

## 2 Results and Discussion

To identify the optimal conditions for the oxidative transformation of levulinic acid **1** to citramalic acid **2**, we systematically evaluated the effects of solvent, base, temperature, and reaction atmosphere (Table 1). Our initial investigation focused on the influence of solvent choice under ambient temperature, employing KO*t*Bu (3.0 equiv.) under an oxygen atmosphere. Tetrahydrofuran (THF), a polar aprotic solvent, afforded citramalic acid **2** along with a mixture of succinic acid **3**, acetic acid **A**, and formic acid **B**, resulting in 41% conversion, whereas 1,4‐dioxane led to only 17% conversion with no detectable formation of **2**, **3**, **A**, or **B** (entries 1–2).

**TABLE 1:** Optimization of reaction conditions.^a^

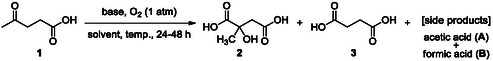						
Entry	Solvent (M)	Base (eq.)	Time, h	Temp, °C	Conv., %^d^	Ratio of **2**:**3**:**A**:**B** d
1	THF [0.3]	KO*t*Bu [3]	48	RT	41	12 : 2 : 10 : 6
2	1,4‐dioxane [0.3]	KO*t*Bu [3]	48	RT	17	n.d : n.d : n.d : n.d
3	*n*‐heptane [0.3]	KO*t*Bu [3]	48	RT	19	9 : 1 : 6 : 3
4	Toluene [0.3]	KO*t*Bu [3]	48	RT	16	7 : 1 : 5 : 2
5	Dichloromethane [0.3]	KO*t*Bu [3]	48	RT	11	3 : n.d : 1 : 7
6	MeOH [0.3]	KO*t*Bu [3]	48	RT	0	n.d : n.d : n.d : n.d
7	*n*‐BuOH [0.3]	KO*t*Bu [3]	48	RT	0	n.d : n.d : n.d : n.d
8	*iso*‐PrOH [0.3]	KO*t*Bu [3]	48	RT	18	4 : 1 : 2 : 1
9	*t*‐BuOH [0.3]	KO*t*Bu [3]	48	RT	61	47 : 1 : 6 : 3
10	*t*‐BuOH [0.3]	KO*t*Bu [3]	48	30	98	91 : 1 : n.d : n.d
11	*t*‐BuOH [0.3]	KO*t*Bu [3]	24	30	95	61 : 1 : 31 : 6
12^b^	*t*‐BuOH [0.3]	KO*t*Bu [3]	48	30	0	n.d : n.d : n.d : n.d
13	*t*‐BuOH [0.3]	—	48	30	0	n.d : n.d : n.d : n.d
14	*t*‐BuOH [0.3]	KO*t*Bu [2]	48	30	72	29 : 1 : 10 : 2
15	*t*‐BuOH [0.3]	KO*t*Bu [4]	48	30	98	48 : 1 : 19 : 7
16	*t*‐BuOH [0.3]	KOMe [3]	48	30	87	16 : 1 : 8 : 22
17	*t*‐BuOH [0.3]	KOH [3]	48	30	86	17 : 3 : 8 : 1
18	*t*‐BuOH [0.3]	NaO*t*Bu [3]	48	30	48	40 : 1 : 13 : 4
19	*t*‐BuOH [0.3]	LiO*t*Bu [3]	48	30	54	18 : 3 : 2 : 6
20^c^	*t*‐BuOH [0.3]	KO*t*Bu [3]	48	30	24	7 : 1 : 4 : 8

a The reaction was carried out with levulinic acid **1** (1 mmol), base (3–4 equiv.), solvent (0.3 M) for 24–48 h under an oxygen atmosphere (1 atm).

b The reaction was carried out under argon atmosphere.

c The reaction was carried out under aerobic conditions using air instead of pure oxygen.

d The conversions and yield ratio of the products were determined by ^1^H NMR spectroscopy using maleic acid as an internal standard.

The formation of side products **A** and **B** was confirmed by NMR spectroscopy, and a detailed reaction mechanism is provided in the Supporting Information. Nonpolar solvents such as *n*‐heptane and toluene gave yields comparable to those observed in 1,4‐dioxane but resulted in the formation of **2**, **3**, **4**, and **5** (entries 3–4). The conversion observed in dichloromethane was similarly low (11%), indicating that solvent polarity alone is insufficient to promote the transformation (entry 5). Alcoholic solvents such as methanol and *n*‐butanol completely inhibited the reaction (entries 6–7), whereas isopropanol provided only marginal improvement (18% conversion, entry 8).

**SCHEME 2:**
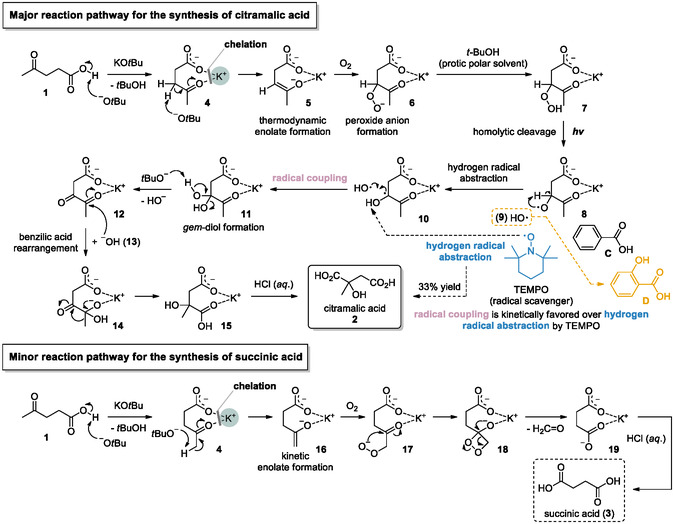
A distinct proposed reaction mechanism accounts for the formation of citramalic acid as the major product and succinic acid as the minor product.

By switching the solvent from isopropanol to *t*‐butanol at room temperature, both the conversion and the selectivity between compounds **2** and **3** were significantly improved (entry 9). To further optimize both conversion and selectivity, the reaction temperature was elevated, resulting in 98% conversion and a 91:1 selectivity for citramalic acid **2** over succinic acid **3** at 30ºC (entry 10). In contrast, a deterioration in selectivity was observed at shorter reaction times (24 h, entry 11), thereby establishing the use of *t*‐BuOH at 30°C for 48 h as the optimal condition. Notably, both reaction temperature and time are critical parameters in this study, playing key roles in the reaction mechanism, as emphasized in Scheme 2.

Control experiments unequivocally established the indispensable roles of both the base and oxygen. Under anaerobic conditions, no conversion was detected (entry 12), and likewise, the absence of base resulted in complete inactivity (entry 13). These findings clearly demonstrate the dual necessity of KO*t*Bu and O_2_ for the reaction to proceed. We subsequently examined the effect of varying the equivalents of KO*t*Bu. Reducing the base to 2.0 equivalents decreased the conversion to 72% (entry 14), whereas increasing it to 4.0 equivalents maintained a high conversion of 98% (entry 15) but led to diminished selectivity toward the desired product. Collectively, these results indicate that 3.0 equivalents of KO*t*Bu strike an optimal balance between reactivity and atom economy, being both sufficient and efficient.

Next, a base screen was conducted to evaluate the influence of base identity on the reaction outcome (entries 16–19). Both KOMe and KOH afforded respectable conversions of 87% and 86%, respectively, albeit with lower selectivity compared to KO*t*Bu (entries 16 and 17). In contrast, the markedly lower conversions observed with NaO*t*Bu and LiO*t*Bu attest to the crucial role of the metal cation in modulating both reactivity and selectivity (entries 18 and 19). When the reaction was conducted under aerobic conditions using air instead of pure oxygen, the conversion dropped to 24%, attributable to the lower oxygen content in air, which significantly impacts reaction efficiency (entry 20).

Based on these observations, particularly the preferential formation of citramalic acid **2** over succinic acid **3**, we postulated the underlying reaction mechanism, as illustrated in Scheme 2.

**FIGURE 1:**
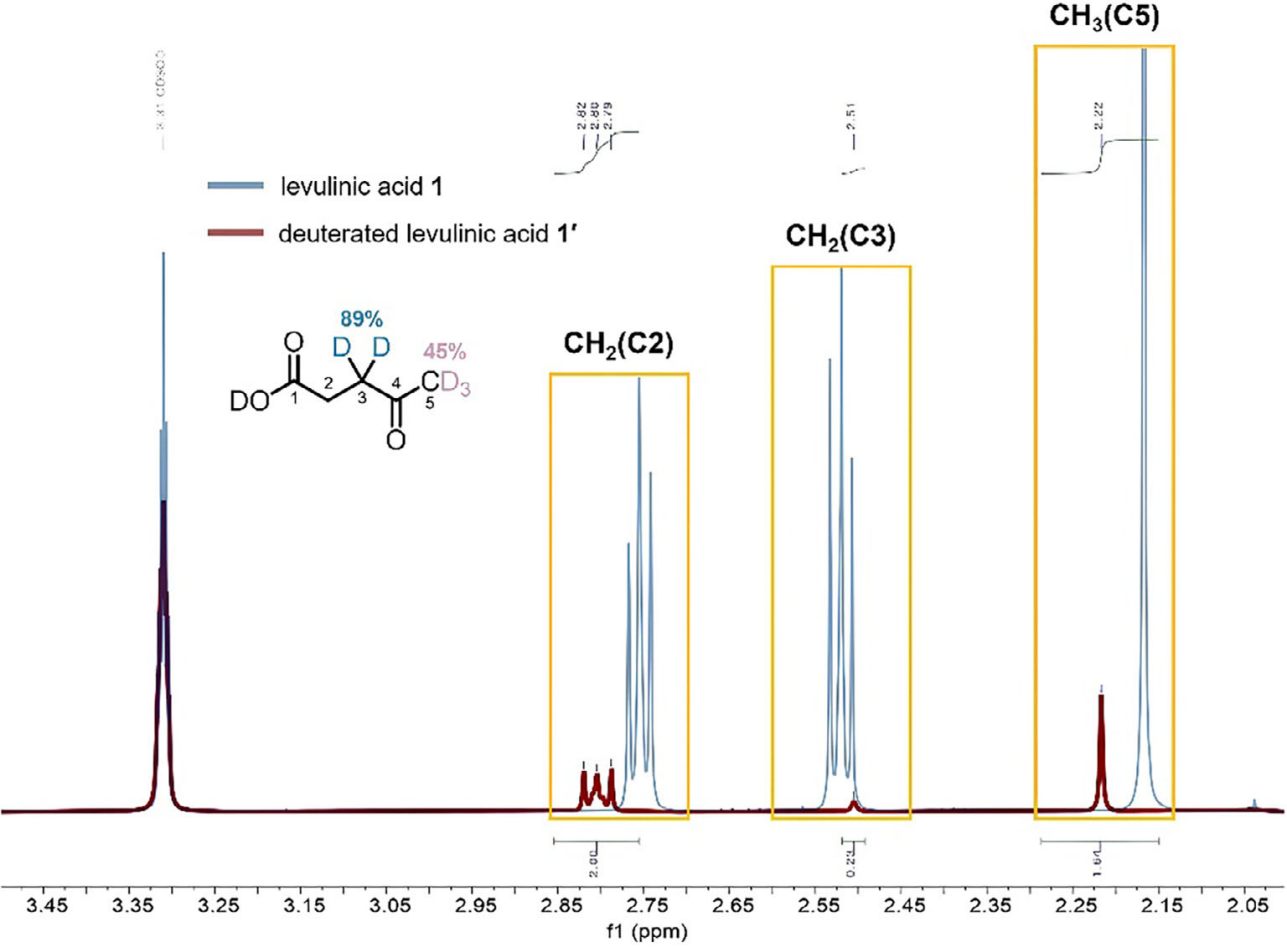
Deuterium‐labeling experiment demonstrating the formation of the thermodynamic enolate and the ^1^H NMR spectrum of deuterated levulinic acid (**1′**). Reaction conditions for deuterium labeling: levulinic acid **1** (1.0 mmol) was treated with KO*t*Bu (3.0 equiv.) in THF (0.3 M) at room temperature for 3 h under an Ar atmosphere, followed by the addition of D_2_O (3.0 mL) and stirring for 30 min prior to quenching with 1 N aqueous HCl (for details, see the Supporting Information).

Potassium *t*‐butoxide initially serves as a base to abstract a proton from levulinic acid **1**, resulting in complexation with the K^+^ cation (**4**) and the concomitant release of *t*‐butanol [26, 27]. Notably, the complexation between the carbonyl groups of levulinic acid and the K^+^ cation acts as a promoter, primarily due to the optimal fit of the K^+^ ionic radius, which is presumably better suited than those of Li^+^ or Na^+^ to facilitate effective coordination, thereby exerting a significant influence on both reactivity and conversion (see Table 1, entries 18–19). A pivotal step involves the atypical behavior of a free *t*‐butoxide anion: despite its usual preference for kinetic enolate formation **5** due to significant steric hindrance, it can function as a thermodynamically favored base under equilibrating conditions—such as elevated temperatures and extended reaction times—thereby facilitating the selective formation of the thermodynamic enolate via abstraction of an *α*‐proton from the K^+^–coordinated carbonyl complex [27]. The unusual role of *t*‐butoxide in promoting the formation of the thermodynamic enolate from levulinic acid **1** through carbonyl deprotonation is unambiguously demonstrated by deuterium‐labeling experiments and further corroborated by NMR spectroscopic analysis (Figure 1). The near disappearance of the two CH_2_ protons at the C3 position (2.52 ppm) in the NMR spectra after deuterium‐labeling provides strong evidence for the formation of the thermodynamic enolate rather than the kinetic enolate. For precise assessment of deuterium incorporation in elucidating the thermodynamic enolate formation pathway, the same solvent system (e.g., *t*‐BuOH) would ideally be employed, as it represents the optimal conditions. However, in the protic polar solvent *t*‐BuOH, deuterium incorporation was also observed at the C5 methyl group, indicative of concomitant kinetic enolate formation. To minimize deuterium incorporation at the C5 position, THF, an aprotic polar solvent, was therefore employed at room temperature. Although these deviations from the optimal reaction parameters led to partial formation of the kinetic enolate, the deuterium‐labeling experiments collectively confirm that the thermodynamic enolate remains the predominant intermediate. Furthermore, the theoretical expectation that the equilibrium between the kinetic and thermodynamic enolates shifts toward the thermodynamic enolate at elevated temperatures is fully consistent with the experimental results: at 30°C (Table 1, entry 10), the thermodynamic enolate is formed predominantly compared to reactions conducted at room temperature (Table 1, entry 9).

**SCHEME 3:**
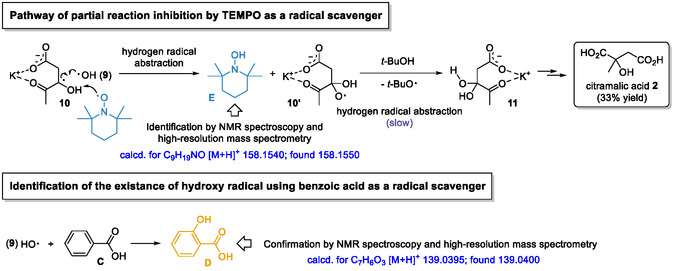
Mechanistic pathways of radical scavenging by TEMPO and benzoic acid.

The resulting thermodynamic enolate promptly reacts with molecular oxygen to form an *α*‐peroxy anion **6** adjacent to the carbonyl group. This intermediate subsequently undergoes protonation in the presence of excess *t*‐butanol, which serves as the proton source and plays a crucial role in citramalic acid formation. The mechanistic rationale for this transformation is outlined as follows. i) the hydroperoxide moiety **7** rapidly undergoes homolytic cleavage of its O–O bond under light irradiation, generating two distinct oxygen‐centered radical species (8 and 9) [28, 29]. Under light‐shielded (dark) conditions, the yield of the desired citramalic acid **2** decreased markedly to 50%, compared with the optimal yield of 91% (Table 1, entry 10), indicating that light irradiation is crucial for generating two distinct oxygen‐centered radical species (**8** and **9**), which collectively exert a significant influence on the overall chemical yield. The liberating hydroxyl radical **9** was confirmed using benzoic acid (**C**) as a radical scavenger, which resulted in the formation of salicylic acid (**D**) [30]. In addition, the introduction of TEMPO as a radical scavenger led to the detection of TEMPO‐H (**E**) [31]. Consistent with radical interception, TEMPO partially suppressed the formation of the desired citramalic acid, affording a reduced yield of 33% [32]. The preserved yield of 33% nonetheless suggests that radical coupling proceeds at a comparatively higher kinetic rate than the competing TEMPO‐mediated hydrogen abstraction from intermediate **10**. The detailed reaction mechanism is illustrated in Scheme 3. ii) The formation of *gem*‐diol **11**, a direct precursor to diketone **12**, proceeds through hydrogen‐atom abstraction at the same carbon center of radical **8** by an oxygen‐centered radical, which initiates a [1,2]‐migration [33]. The resulting carbon‐centered radical (**10**) subsequently couples with the liberated hydroxyl radical **9**, thereby completing the transformation [34].

Under basic conditions, the resulting *gem*‐diol **11** readily converts into the diketone **12** with the concomitant release of hydroxide ion **13** [35, 36]. This intermediate subsequently undergoes a typical benzilic acid rearrangement, leading to the formation of intermediate **14**, which incorporates methyl and hydroxyl groups through the development of a tetrahedral geometry [37, 38, 39, 40, 41, 42, 43, 44, 45]. Upon acidic aqueous work‐up of the intermediate **15**, the desired citramalic acid **2** is obtained efficiently.

In contrast to polar protic solvents such as *t*‐butanol, nonpolar aprotic solvents like *n*‐heptane and toluene generally provide a low‐dielectric environment that can hinder both the generation and stabilization of radical intermediates [46]. As a consequence of these solvent properties, diminished product yields were observed (Table 1, entries 3–4). Similarly, the use of dichloromethane, despite being more polar than *n*‐heptane and toluene, also resulted in reduced yield (Table 1, entry 5). This decrease is likely due to the absence of a suitable proton source required for the formation of a hydrogen peroxide intermediate, which undergoes homolytic cleavage followed by subsequent transformations en route to the desired product **2**.

The proposed reaction mechanism for the formation of succinic acid **3** as a minor byproduct is outlined below. In contrast to the formation of thermodynamic enolate **5**, which represents the key step in the pathway toward citramalic acid synthesis, kinetic enolate **16** is generated as a competing minor pathway. Under an oxygen atmosphere, the kinetic enolate **16** can undergo *α*‐functionalization to form an *α*‐peroxy anion **17**, which subsequently participates in intramolecular nucleophilic addition to the adjacent carbonyl group, affording the cyclized intermediate **18**. The highly strained dioxetaneolate **18** readily undergoes ring opening to generate intermediate **19**, which ultimately furnishes succinic acid **3** as a minor product upon acidic aqueous work‐up.

Detailed information on the initial radical‐trapping process is provided in Scheme 3. TEMPO, acting as a radical scavenger, abstracts a hydrogen radical from intermediate **10** to form TEMPO‐H (**E**), as confirmed by NMR spectroscopy and high‐resolution mass spectrometry (HRMS). The resulting intermediate **10′** subsequently undergoes hydrogen radical abstraction from *t*‐BuOH, a step that constitutes the rate‐limiting process. Despite the presence of radical species, hydrogen abstraction from *t*‐BuOH is intrinsically slow owing to the high bond dissociation energy of the O–H bond, substantial steric hindrance at the abstraction site, and the limited stabilization of the resulting radical. This rate‐retarding step strongly influences the formation of *gem*‐diol **11**, consistent with the observed 33% yield of the desired product **2**. Furthermore, the hydroxyl radical **9**, generated from **7** via light‐induced homolytic cleavage, was detected using benzoic acid (**C**) as a hydroxyl radical scavenger. The resulting formation of salicylic acid (**D**) was confirmed by NMR spectroscopy and HRMS.

**SCHEME 4:**
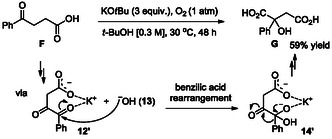
Solid evidence for the involvement of hydroxide ion in the formation of product **G** via the benzilic acid rearrangement.

To ascertain the presence of hydroxide ion **13** as a key species in the benzilic acid rearrangement of 1,2‐diketone (**12**), we employed 4‐oxo‐4‐phenylbutanoic acid (**F**) as an analogue of levulinic acid **1** under optimized conditions (Scheme 4). The successful formation of 2‐hydroxy‐2‐phenylsuccinic acid (**G**) via benzilic acid rearrangement from **12′** to **14′** confirms the involvement of hydroxide ion **13** as a crucial factor in the reaction mechanism, despite not being externally introduced into the system (Scheme 4). This evidence for the participation of hydroxide ion **13** strongly supports the proposed reaction mechanisms and further deepens the mechanistic understanding, as depicted in Scheme 2.

**SCHEME 5:**
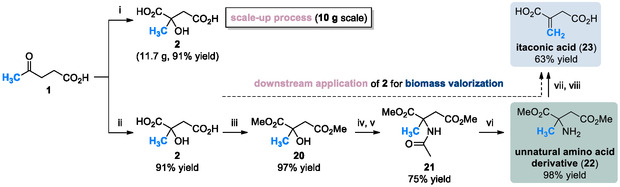
Citramalic acid **2** as a platform chemical for scalable synthesis from levulinic acid **1** and its downstream access to the unnatural amino acid derivative **22** and itaconic acid **23**. Reagents and conditions: (i) levulinic acid **1** (10.0 g, 86.2 mmol), KO*t*Bu (3.0 equiv.), *t*BuOH (0.25 M), O_2_ (1 atm), 40°C, 72 h, 91%; (ii) levulinic acid **1** (116.1 mg, 1.0 mmol), KO*t*Bu (3.0 equiv.), *t*BuOH (0.3 M), O_2_ (1 atm), 30°C, 48 h, 91%; (iii) citramalic acid **2** (148.1 mg, 1.0 mmol), SOCl_2_ (2.0 equiv.), MeOH (2.0 M), 0°C to RT, 3 h, 97%; (iv) methyl citramalate **20** (176.2 mg, 1.0 mmol), H_2_SO_4_ (cat.), CH_3_CN (0.2 M), reflux, 6 h, followed by (v) H_2_O, 3 h, RT, 75%; (vi) dimethyl 2‐acetamido‐2‐methylsuccinate **21** (217.2 mg, 1.0 mmol), Na (3.0 equiv.), MeOH (0.2 M), reflux, 6 h, 98%; (vii) dimethyl 2‐amino‐2‐methylsuccinate **22** (175.2 mg, 1.0 mmol), K_2_CO_3_ (1.0 equiv.), MeI (excess, 5.0 equiv.), MeOH (0.2 M), followed by evaporation of the methanol solvent and treatment with (viii) K_2_CO_3_ (3.0 equiv.), H_2_O/CH_3_CN (v/v = 1:2, 0.2 M), 80°C, 6 h, 63%.

We further evaluated the selective oxidation of levulinic acid **1** to citramalic acid **2** as a potential chemical platform, exploring its scalability and downstream transformations (Scheme 5). First, the reaction was successfully performed on a 10 g scale, representing an 86‐fold increase relative to the scale described in Table 1, without modification of the optimized conditions. This direct scalability highlights not only the practical utility of chemistry but also its compatibility with sustainable process development. Importantly, the present transformation is not catalytic but base‐mediated. Stoichiometric KO*t*Bu serves a dual role as both a base for enolate generation and a mediator of the radical oxygenation process under molecular oxygen. Upon completion of the reaction, KO*t*Bu is converted into *t*‐BuOH, which cannot be readily regenerated or recycled as the active base without additional energy‐intensive treatment; therefore, the recycling of KO*t*Bu was not feasible. Nevertheless, the sustainability of this method arises from the complete elimination of transition metals and pressurized oxidants, the use of inexpensive and commercially available reagents, and the mild reaction conditions that enable high selectivity and scalability.

Second, the downstream application of citramalic acid **2** in the synthesis of an unnatural amino acid ester **22** proceeds as follows. Unnatural amino acid diester **22** was synthesized from levulinic acid **1** via esterification with thionyl chloride in methanol, followed by a Ritter reaction using acetonitrile as the nitrogen source, and subsequent deacetylation of intermediate **21** using sodium in methanol. To the best of our knowledge, this transformation is unprecedented, as no prior chemical method has enabled the direct preparation of this class of amino acid derivatives **22** from a renewable feedstock. Third, the resulting unnatural amino acid diester **22** was efficiently converted into itaconic acid **23** via a Hofmann elimination. Itaconic acid **23** is a valuable C5 unsaturated diacid widely recognized for its versatility in the production of bio‐based polymers and specialty chemicals. Although itaconic acid **23** is predominantly produced through microbial fermentation, our approach provides a rare and efficient chemical alternative by enabling the installation of a terminal olefinic group through a straightforward Hofmann elimination, thereby eliminating the need for microbial fermentation or transition‐metal catalysis. Overall, the progression from levulinic acid **1** to citramalic acid **2**, and subsequently to unnatural amino acid diester **22** and itaconic acid **23**, establishes a novel synthetic paradigm that extends the significance of this platform beyond conventional biotransformations.

## 3 Conclusion

In summary, we have developed a direct and operationally simple oxidation of levulinic acid to citramalic acid using a KO*t*Bu–O_2_ system that completely avoids microbial fermentation as well as transition metals or engineered heterogeneous catalysts. By operating under mild conditions with readily available reagents, this approach provides a sustainable chemical alternative that overcomes key limitations associated with biotechnological and metal‐catalyzed routes. This metal‐free process not only offers a scalable and sustainable strategy for biomass upgrading but also reveals an unexpected facet of enolate chemistry, governed by the cooperative interplay of enolate equilibration, radical intermediacy, and base–substrate organization. The robustness of the transformation, validated on the decigram‐to‐decagram scale, attests to its practical relevance for sustainable chemical manufacturing. Recognizing citramalic acid as a multifunctional platform molecule relevant to sustainable biofuels, biodegradable polymers, and specialty chemicals, we further demonstrate its downstream valorization through selective molecular editing, including conversion to an unnatural amino acid ester via a Ritter reaction and transformation into itaconic acid through Hofmann elimination. These derivatizations illustrate the strategic exploitation of the intrinsic reactivity of citramalic acid to access structurally diverse and industrially relevant products, thereby extending its utility beyond classical biotransformations. More broadly, this work integrates mechanistic insight with process‐oriented synthetic design to establish a sustainable reaction platform for oxidative C–C functionalization. Although the system is noncatalytic, its metal‐free nature and operational simplicity provide a practical and sustainable pathway for biomass valorization. Taken together, these findings demonstrate how a simple yet powerful chemical strategy can expand the methodological space of oxidative C–C functionalization and open new opportunities for the cost‐effective valorization of widely available biomass‐derived feedstocks.

## Supporting Information

Additional supporting information can be found online in the Supporting Information section. **Supporting** **Scheme S1:** Mechanistic rationale for by‐product formation in the synthesis of citramalic acid.

## Funding

This work was supported by National Research Foundation of Korea (NRF) (RS‐2023‐NR076432, RS‐2023‐00219859), and Korea Basic Science Institute (National Research Facilities and Equipment Center) (RS‐2022‐NF000854, RS‐2025‐02413546).

## Conflicts of Interest

The authors declare no conflicts of interest.

## Supporting information

Supplementary Material

## Data Availability

The data that support the ﬁndings of this study are available in the supplementary material of this article.
