# Supplementary material for: An Unorthodox Enolate–Triggered Radical Relay Directs the Chemo Upgrading of Levulinic Acid Into Citramalic Acid
Source: ChemSusChem. 2026 Feb 24;19(4):e202502757. doi: 10.1002/cssc.202502757 (PMC12932077; doi:10.1002/cssc.202502757)

Supporting Information

An Unorthodox Enolate–Triggered Radical Relay Directs the Chemo-Upgrading of Levulinic Acid into Citramalic Acid

Geun Ho Kim[a]†, Young Kwang Han[a]†, Tae Woo Lee[a], Eun Jeong Yoo[b], and Jung Woon Yang[a]*

[a] Department of Energy Science, Sungkyunkwan University, Suwon 16419, Republic of Korea
[b] Department of Applied Chemistry, Kyung Hee University, Yongin 17104, Republic of Korea
E-mail address: jwyang@skku.edu (J. W. Yang).

† These authors contributed equally to this work.

Dedicated to Professor Chulbom Lee on the occasion of his 60th birthday

Contents

General Remarks and Experimental Procedures S2
Characterization Data for the Products S6
References S9
^{1}H NMR and ^{13}C NMR Spectra of the Products S10

General Remarks

Commercial-grade reagents and solvents were used without further purification. Thin-layer chromatography (TLC) was performed on Merck Kieselgel silica gel 60 F254. Flash chromatography was carried out using Merck silica gel (60 Å, 230−400 mesh, particle size 0.040−0.063 mm). ^{1}H and ^{13}C nuclear magnetic resonance (NMR) spectra were recorded on a Bruker Ascend™ 500 spectrometer in a suitable deuterated solvent at 298 K. Chemical shift values are reported in ppm relative to tetramethylsilane as the internal standard, with one or two digits after the decimal point. The product yield from the general procedure was determined by ^{1}H NMR spectroscopy using maleic acid as an internal standard. ^{1}H NMR spectra were recorded at 500 MHz in $CDCl_3$ (δ 7.26 ppm), D_2O (δ 4.79 ppm), or CD_3OD (δ 3.31 ppm). ^{13}C NMR spectra were recorded at 126 MHz in $CDCl_3$ (δ 77.16 ppm), or CD_3OD (δ 49.00 ppm). The abbreviations m, s, d, t, and q, quint., and sept. denote multiplet, singlet, doublet, triplet, quadruplet, quintuplet, and septet, respectively, and br denotes a broad signal. High-resolution mass spectrometer (HRMS) was performed by electron impact (EI). Mass data were acquired on a supercritical fluid chromatograph combined with a Xevo G2-XS QTOF mass spectrometer (Waters, Milford, MA, USA) at the Chiral Material Core Facility Center of Sungkyunkwan University

Experimental Procedures

General procedure A for the synthesis of citramalic acid 2

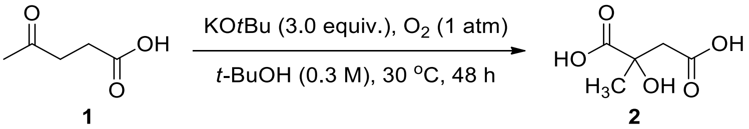

To a stirred solution of levulinic acid **1** (116.1 mg, 1.0 mmol) in *t*-butanol (3.3 mL, 0.3 M), potassium *t*-butoxide (336.6 mg, 3.0 mmol) was added under an oxygen atmosphere at 30 °C. The reaction mixture was stirred for 48 h, after which it was quenched by adjusting the pH to 2 with 1 N aqueous HCl solution. The resulting mixture was filtered through filter paper, and the filtrate was concentrated under reduced pressure using a rotary evaporator to afford citramalic acid **2** as a yellow oil (134.8 mg, 91% yield).

General procedure B for the large-scale synthesis of citramalic acid 2

To a stirred solution of levulinic acid **1** (10.0 g, 86.2 mmol) in *t*-butanol (344 mL, 0.25 M), potassium *t*-butoxide (29.0 g, 258 mmol) was added under an oxygen atmosphere at 40 °C. The reaction mixture was stirred for 72 h. Upon completion, it was quenched by adjusting the pH to 2 with 1 N aqueous HCl solution. The resulting mixture was filtered through filter paper, and the filtrate was concentrated under reduced pressure to afford citramalic acid **2** as a yellow oil (11.7 g, 91% yield).

General procedure C for the synthesis of amino acid ester 22 (Ritter Reaction)

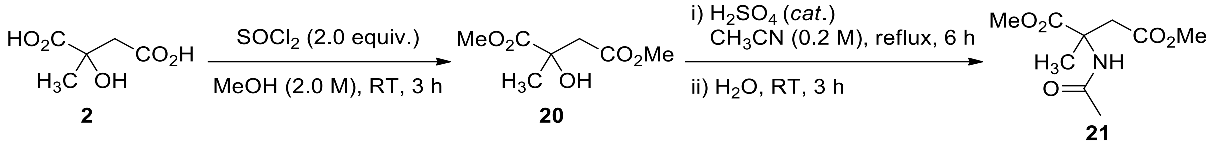

Citramalic acid **2** (148.1 mg, 1.0 mmol) was dissolved in methanol (0.5 mL, 2.0 M), and thionyl chloride (0.15 mL, 2.0 mmol) was added dropwise at 0 °C. The mixture was warmed to room temperature and stirred for 3 h, yielding dimethyl citramalate **20**, which was used directly in the next step without further purification. To a stirred solution of the crude dimethyl citramalate **20** in acetonitrile (5.0 mL, 0.2 M), catalytic H_2SO_4 was added, and the mixture was refluxed for 6 h. The reaction was quenched with water (10 mL) and stirred at room temperature for 3 h to afford the acetamido ester **21** in 75% yield. Without isolation, the crude residue was dissolved in methanol (5.0 mL, 0.2 M), and sodium metal (293.5 mg, 3.0 mmol) was added portionwise. The mixture was refluxed for 6 h under an argon atmosphere, cooled to room temperature, and quenched with saturated NH_4Cl solution. The mixture was extracted with ethyl acetate (3 × 10 mL), and the combined organic layers were dried over anhydrous Na_2SO_4, filtered, and concentrated under reduced pressure. The amino acid ester **22** was obtained in 98% yield without further purification.

General procedure D for the synthesis of itaconic acid 23 (Hofmann elimination)

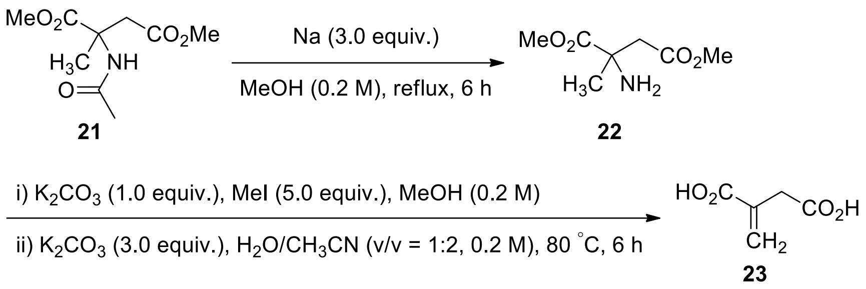

Dimethyl 2-amino-2-methylsuccinate **22** (217.2 mg, 1.0 mmol) was dissolved in methanol (5.0 mL, 0.2 M), and potassium carbonate (138.2 mg, 1.0 mmol) was added, followed by the dropwise addition of excess methyl iodide (0.31 mL, 5.0 mmol) at room temperature. The mixture was stirred until complete consumption of the starting material, as monitored by TLC. The reaction mixture was concentrated under reduced pressure, and the crude residue was used directly in the next step without purification. The residue was dissolved in a mixture of water and acetonitrile (v/v = 1:2, 5.0 mL, 0.2 M), and potassium carbonate (414.6 mg, 3.0 mmol) was added. The mixture was heated to 80 °C and stirred for 6 h. After cooling to room temperature, the reaction mixture was extracted with ethyl acetate (3 × 10 mL) to remove organic byproducts, and the aqueous layer was collected. The water was removed under reduced pressure, and the residue was recrystallized to afford itaconic acid **23** in 63% yield.

General procedure E for the deuterated levulinic acid 1′

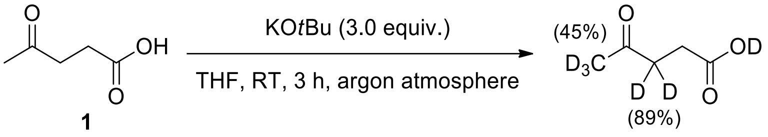

To a stirred solution of levulinic acid **1** (116.1 mg, 1.0 mmol) in THF (3.3 mL, 0.3 M), potassium *t*-butoxide (336.6 mg, 3.0 mmol) was added under an argon atmosphere at room temperature. The resulting suspension was stirred vigorously for 3 hours to ensure complete deprotonation and enolate formation. Subsequently, D_2O (3.0 mL) was added dropwise to the reaction mixture, which was then stirred for 30 min to allow α-deuteration of ketone in levulinic acid. The reaction was quenched by the dropwise addition of 1 N aqueous HCl solution until the mixture reached pH 2. The resulting inorganic solids were removed by filtration through filter paper, and the filtrate was concentrated under reduced pressure using a rotary evaporator to remove residual solvents. The crude residue was then purified by column chromatography with a mixture of MeOH and CH_2Cl_2 (v/v = 5:95) as the eluent, affording deuterated levulinic acid **1′** as the desired product.

General procedure F for the synthesis of 2-hydroxy-2-phenylsuccinic acid G

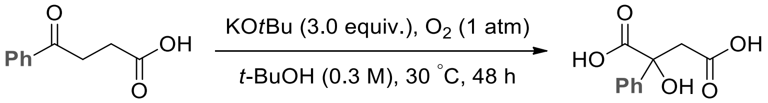

To a stirred solution of 4-oxo-4-phenylbutanoic acid **F** (178.2 mg, 1.0 mmol) in *t*-butanol (3.3 mL, 0.3 M), potassium *t*-butoxide (336.6 mg, 3.0 mmol) was added under an oxygen atmosphere at 30 °C. The reaction mixture was stirred for 48 h, after which it was quenched by adjusting the pH 2 with 1 N aqueous HCl solution. The resulting mixture was filtered through filter paper, and the filtrate was concentrated under reduced pressure using a rotary evaporator to afford 2-hydroxy-2-phenylsuccinic acid **G** as a yellow oil (124.0 mg, 59% yield).

■ Mechanistic rationale for by-product formation: acetic and formic acid in citramalic acid synthesis

Potassium *t*-butoxide initially serves as a strong base to abstract a proton from levulinic acid, generating the corresponding enolate species, which is further stabilized through chelation with the K^+ cation. Upon exposure to molecular oxygen, the resulting intermediate undergoes α-oxygenation at different positions relative to the carbonyl group to form a peroxy species, which then diverges along two distinct competing pathways, each contributing to the formation of by-product.

In the pathway leading to **acetic acid (A)**, the potassium-coordinated enolate reacts with O_2 to generate a peroxy anion at the carbon of thermodynamic enolate species. This intermediate

subsequently adds to carbonyl group to form a dioxetaneolate species, which rapidly undergoes a ring-opening reaction due to ring strain, ultimately yielding the acetic acid (**A**), observed as a minor product.

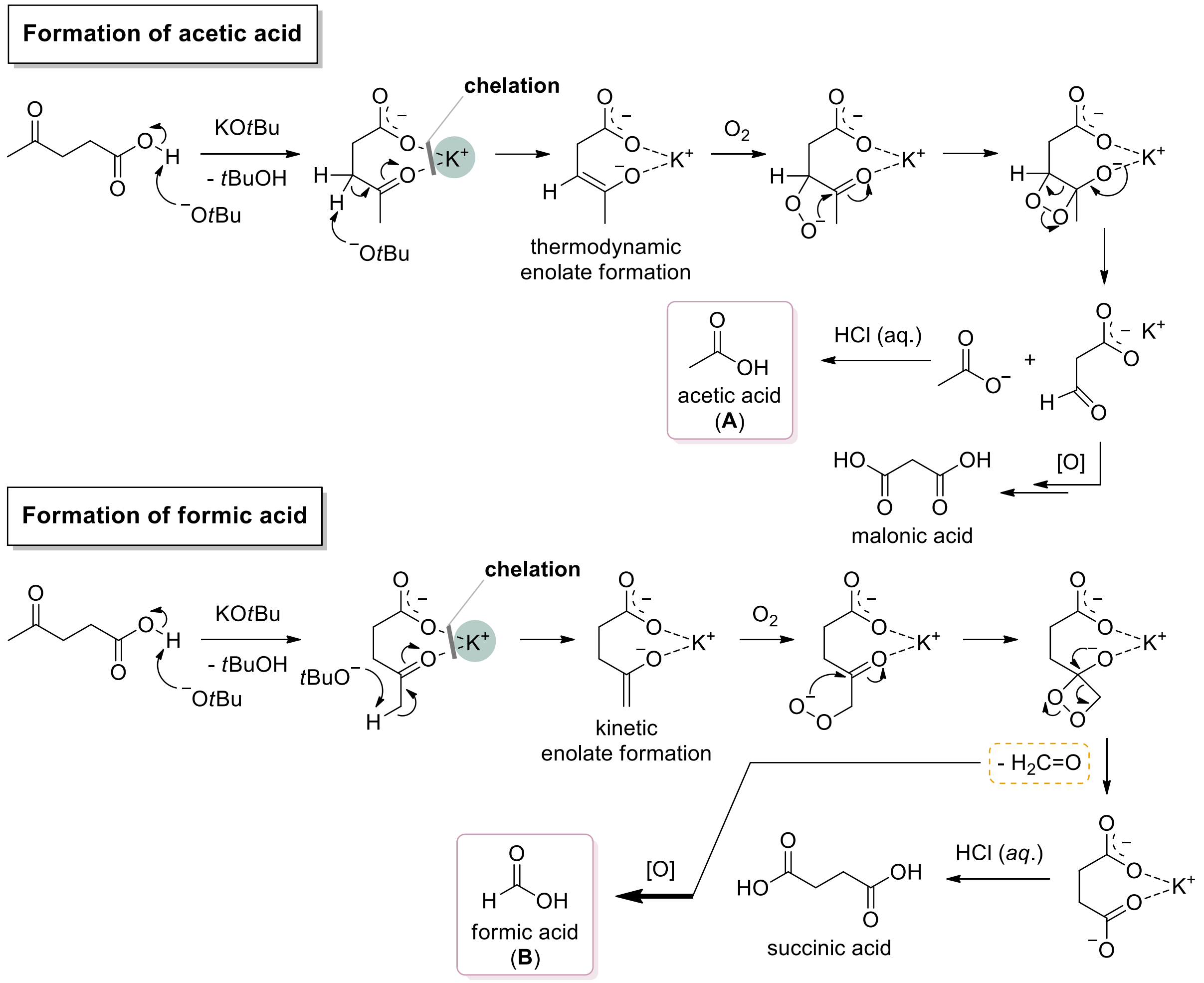

SI Scheme 1. Mechanistic rationale for by-product formation in the synthesis of citramalic acid.

For **formic acid (B)** formation, the mechanistic divergence arises from the initial generation of the kinetic enolate, which, although less thermodynamically favored, can still form under basic conditions. Analogous to the mechanism for acetic acid formation, a dioxetaneolate species is generated through the addition of the peroxy anion to the carbonyl group. This intermediate rapidly undergoes a ring-opening reaction due to ring strain, producing formaldehyde as part of the fragmented products. The resulting formaldehyde is then readily oxidized to formic acid under aerobic oxidation conditions.

Characterization Data for the Products

Deuterated levulinic acid (1′)

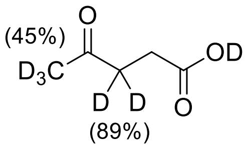

1′ The title compound was synthesized according to general procedure E. Colorless oil. **^{1}H NMR** (500 MHz, CD_3OD) δ 2.84-2.77 (m, 2H), 2.51 (s, 0.23H), 2.22 (s, 1.64H) ppm; **^{13}C NMR** (126 MHz, CD_3OD) δ 209.8, 176.4, 38.7, 29.7, 28.7 ppm. These data were consistent with those reported in the literature.[1,2]

Citramalic acid (2)

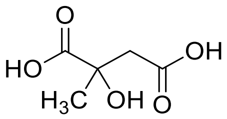

2 The title compound was synthesized according to general procedure A. Reaction time: 48 h, Yield: 91%, Yellow oil. **^{1}H NMR** (500 MHz, D_2O) δ 2.74 (d, J = 15.6 Hz, 1H), 2.43 (d, J = 15.7 Hz, 1H), 1.33 (s, 3H) ppm; **^{13}C NMR** (126 MHz, D_2O) δ 183.1, 179.8, 74.4, 46.6, 25.2 ppm. **HRMS** (ESI, *m/z*): Calcd. for $C_5H_8O_5Na$ $[M+Na]^+$ 171.0269; found 171.0264.

Succinic acid (3)

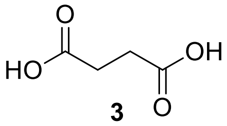

3 The title compound was synthesized according to general procedure A. Reaction time: 48 h, Yield: 1%, White solid. **^{1}H NMR** (500 MHz, D_2O) δ 2.65 (s, 4H) ppm; **^{13}C NMR** (126 MHz, D_2O) δ 177.0, 28.7 ppm. **HRMS** (ESI, *m/z*): Calcd. for $C_4H_6O_4Na$ $[M+Na]^+$ 141.0164; found 141.0158.

Dimethyl citramalate (20)

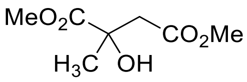

20 The title compound was synthesized according to general procedure C. Reaction time: 3 h, Yield: 97%, White powder. **^{1}H NMR** (500 MHz, $CDCl_3$) δ 3.78 (s, 3H), 3.66 (s, 3H), 2.95 (d, J = 16.5 Hz, 1H), 2.66 (d, J = 16.5 Hz, 1H), 1.42 (s, 3H) ppm; **^{13}C NMR** (126 MHz, $CDCl_3$) δ 176.1, 171.6, 72.6, 53.0, 52.0, 44.1, 26.4 ppm. **HRMS** (ESI, *m/z*): Calcd. for $C_7H_{12}O_5Na$ $[M+Na]^+$ 199.0582; found 199.0583.

Dimethyl 2-acetamido-2-methylsuccinate (21)

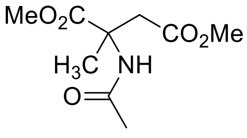

21 The title compound was synthesized according to general procedure C. Reaction time: 9 h, Yield: 75%, Yellow oil. ^{1}**H NMR** (500 MHz, $CDCl_3$) δ 3.67 (s, 3H), 3.61 (s, 3H), 3.07 (d, J = 14.5 Hz, 1H), 2.80 (d, J = 14.3 Hz, 1H), 1.99 (s, 3H), 1.58 (s, 3H) ppm; ^{13}**C NMR** (126 MHz, $CDCl_3$) δ 171.8, 170.0, 169.6, 52.9, 52.0, 40.9, 22.7, 21.1 ppm. **HRMS** (ESI, *m/z*): Calcd. for $C_9H_{15}NO_5Na$ $[M+Na]^+$ 240.0848; found 240.0847.

Dimethyl 2-amino-2-methylsuccinate (22)

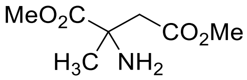

22 The title compound was synthesized according to general procedure C. Reaction time: 6 h, Yield: 98%, Yellow oil. ^{1}**H NMR** (500 MHz, $CDCl_3$) δ 9.60 (br, 2H), 3.77 (s, 3H), 3.75 (s, 3H), 3.00 (d, J = 16.8 Hz, 1H), 2.72 (d, J = 16.8 Hz, 1H), 1.43 (s, 3H) ppm; ^{13}**C NMR** (126 MHz, $CDCl_3$) δ 171.8, 170.8, 59.0, 51.9, 44.0, 26.3 ppm. These data were consistent with those reported in the literature.[3]

Itaconic acid (23)

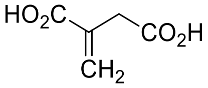

23 The title compound was synthesized according to general procedure D. Reaction time: 6 h, Yield: 63%, White powder. ^{1}**H NMR** (500 MHz, D_2O) δ 6.35 (s, 1H), 5.87 (d, J = 0.9 Hz, 1H), 3.40 (d, J = 1.1 Hz, 2H) ppm; ^{13}**C NMR** (126 MHz, D_2O) δ 175.8, 169.9, 133.4, 130.7, 37.3 ppm. **HRMS** (ESI, *m/z*): Calcd. for $C_5H_6O_4Na$ $[M+Na]^+$ 153.0164; found 153.0167.

Acetic acid (A)

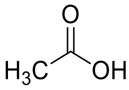

A The title compound was synthesized according to SI scheme 1. Colorless liquid. ^{1}**H NMR** (500 MHz, D_2O) δ 2.03 (s, 3H) ppm; ^{13}**C NMR** (126 MHz, D_2O) δ 176.6, 20.3 ppm. These data were consistent with those reported in the literature.[4]

Formic acid (B)

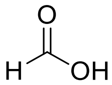

B The title compound was synthesized according to SI Scheme 1. Colorless liquid. ^{1}**H NMR** (500 MHz, D_2O) δ 8.11 (s, 1H) ppm; ^{13}**C NMR** (126 MHz, D_2O) δ 165.6 ppm. These data were consistent with those reported in the literature.[5]

Salicylic acid (D)

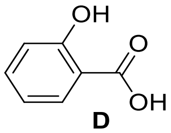

D The title compound was synthesized according to Scheme 2. White solid. **^{1}H NMR** (500 MHz, $CDCl_3$) δ 10.35 (s, 1H), 7.94 (dd, J = 7.9, 1.8 Hz, 1H), 7.54 (ddd, J = 8.7, 7.2, 1.8 Hz, 1H), 7.02 (dd, J = 8.4, 1.2 Hz, 1H), 6.95 (ddd, J = 8.2, 7.1, 1.1 Hz, 1H) ppm; **^{13}C NMR** (126 MHz, $CDCl_3$) δ 175.1, 162.4, 137.2, 131.1, 119.8, 118.0, 111.4 ppm. **HRMS** (ESI, *m/z*): Calcd. for $C_7H_7O_3$ $[M+H]^+$ 139.0395; found 139.0400.

2,2,6,6-Tetramethylpiperidin-1-ol [TEMPO-H (E)]

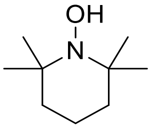

E The title compound was synthesized according to Scheme 2. Yellow oil. **^{1}H NMR** (500 MHz, $CDCl_3$) δ 4.37 (br, 1H), 1.58 (s, 6H), 1.22 (s, 12H) ppm; **^{13}C NMR** (126 MHz, $CDCl_3$) δ 58.6, 38.3, 28.6, 16.0 ppm. **HRMS** (ESI, *m/z*): Calcd. for $C_9H_{19}NO$ $[M+H]^+$ 158.1540; found 158.1550.

2-Hydroxy-2-phenylsuccinic acid (G)

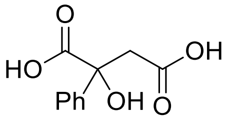

G The title compound was synthesized according to Scheme 4. Yellow oil. **^{1}H NMR** (500 MHz, $CDCl_3$) δ 7.52 – 7.43 (m, 2H), 7.37 – 7.33 (m, 2H), 7.29 (m, 1H), 3.44 (d, J = 16.6 Hz, 1H), 2.89 (d, J = 16.6 Hz, 1H) ppm; **^{13}C NMR** (126 MHz, $CDCl_3$) δ 174.4, 171.8, 140.2, 128.4, 128.2, 126.6, 76.4, 44.4 ppm. These data were consistent with those reported in the literature.[6]

References

[1] A. W. Nørgaard, A. Vibenholt, M. Benassi, P. A. Clausen, P. Wolkoff, *J. Am. Soc. Mass Spectrom.* **2013**, *24*, 1090–1096.

[2] A. A. Scholte, J. C. Vederas, *Org. Biomol. Chem.* **2006**, *4*, 730–742.

[3] A. Amer, *Zagazig J. Pharm. Sci.* **1994**, *3*, 113–118.

[4] M. C. Letzel, B. Decker, A. B. Rozhenko, W. W. Schoeller, J. Mattay, *J. Am. Chem. Soc.* **2004**, *126*, 9669–9674.

[5] P. Haack, A. Kärgel, C. Greco, J. Dokic, B. Braun, F. F. Pfaff, S. Mebs, K. Ray, C. Limberg, *J. Am. Chem. Soc.* **2013**, *135* 16148–16160.

[6] K.-F. Hebenbrock, *Justus Liebigs Ann. Chem.* **1978**, *1978*, 320–336.

^{1}H NMR and ^{13}C NMR Spectra of the Products

Deuterated levulinic acid (1′)

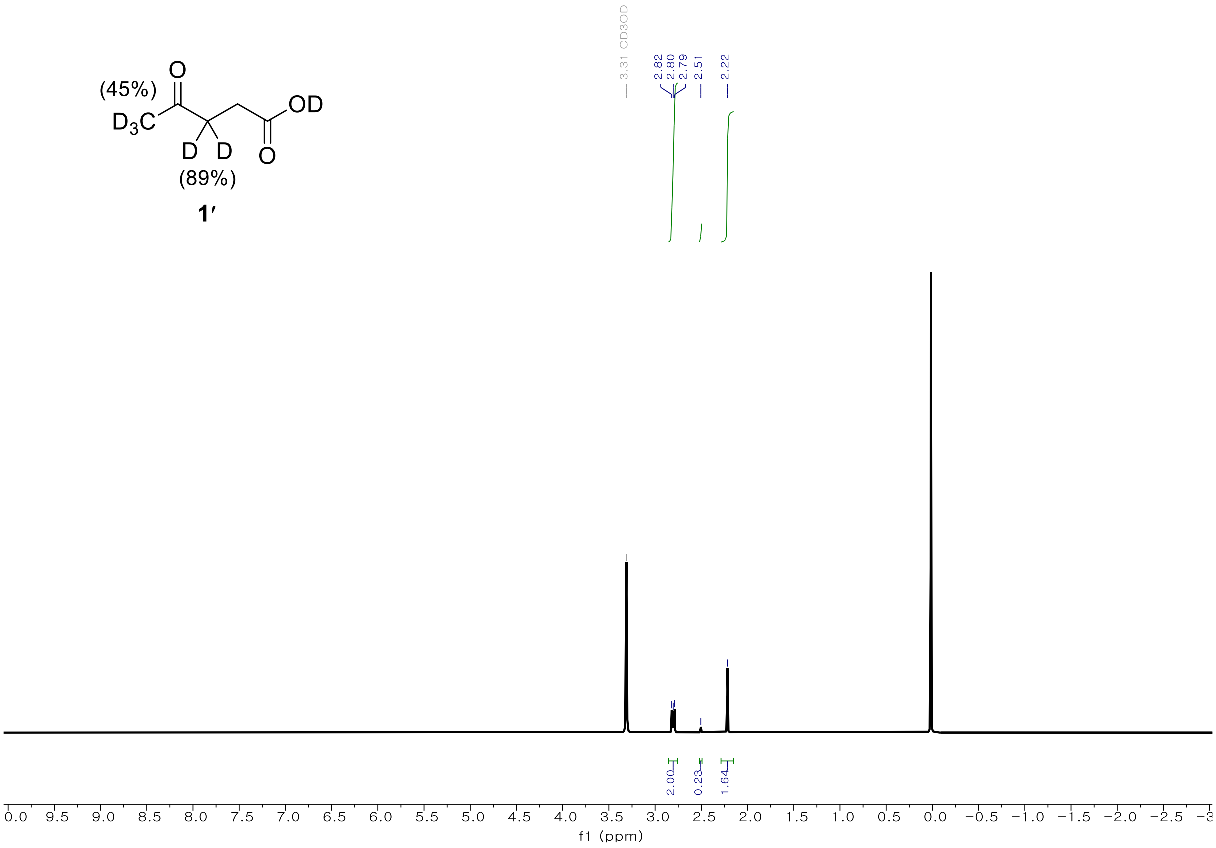

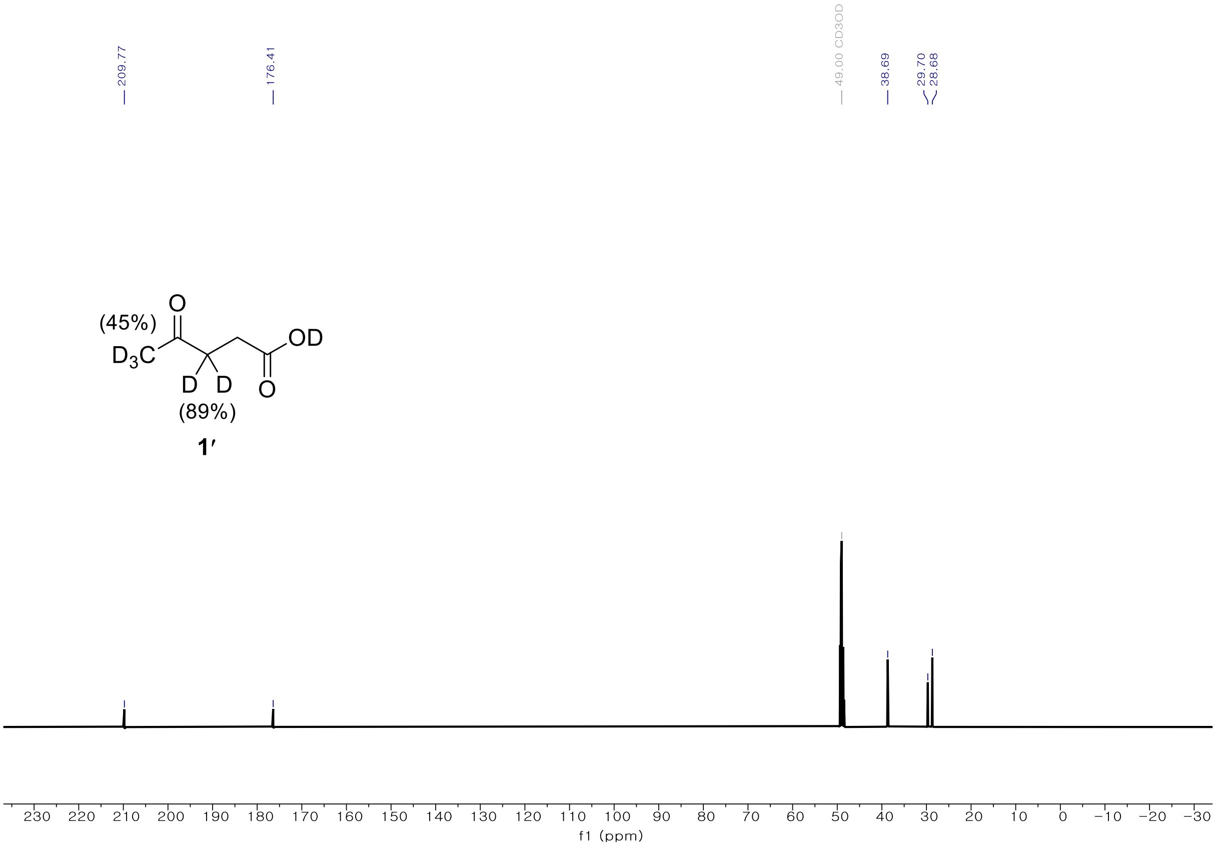

Citramalic acid (2)

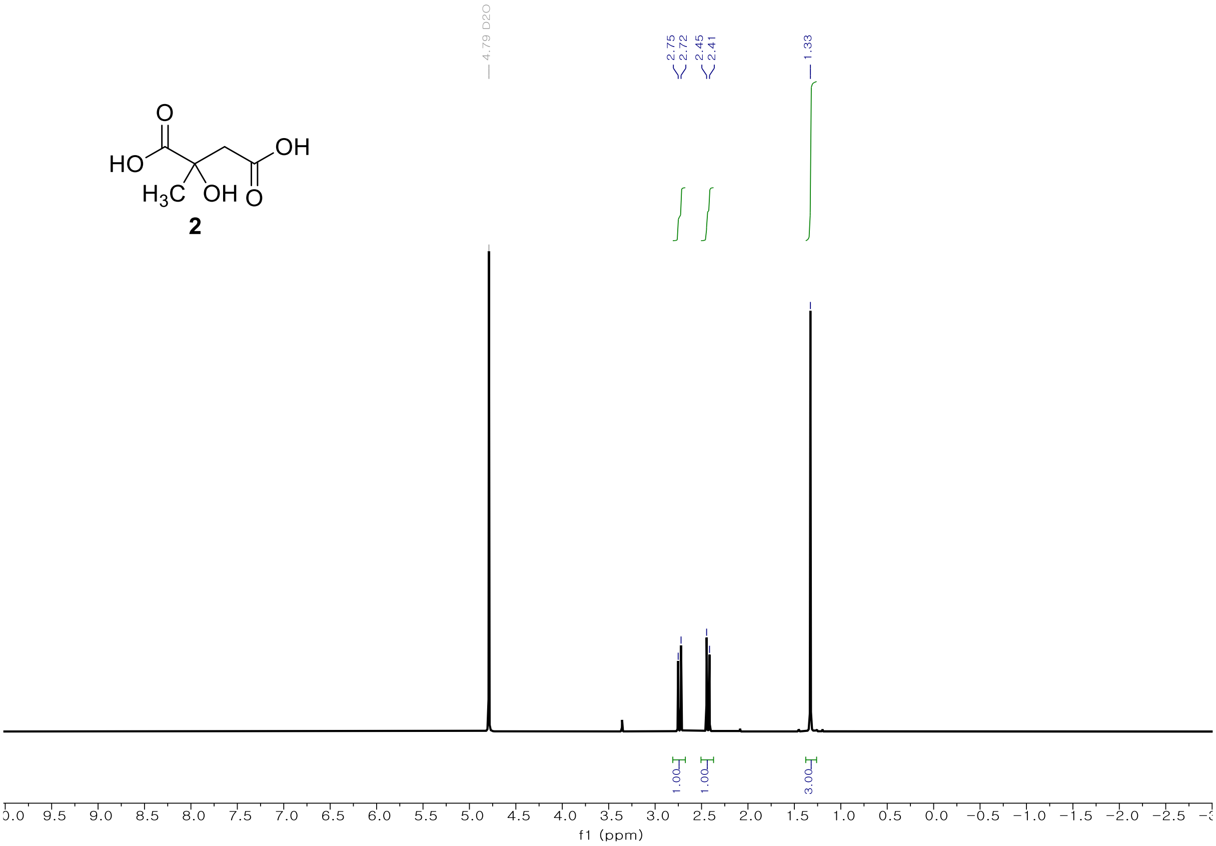

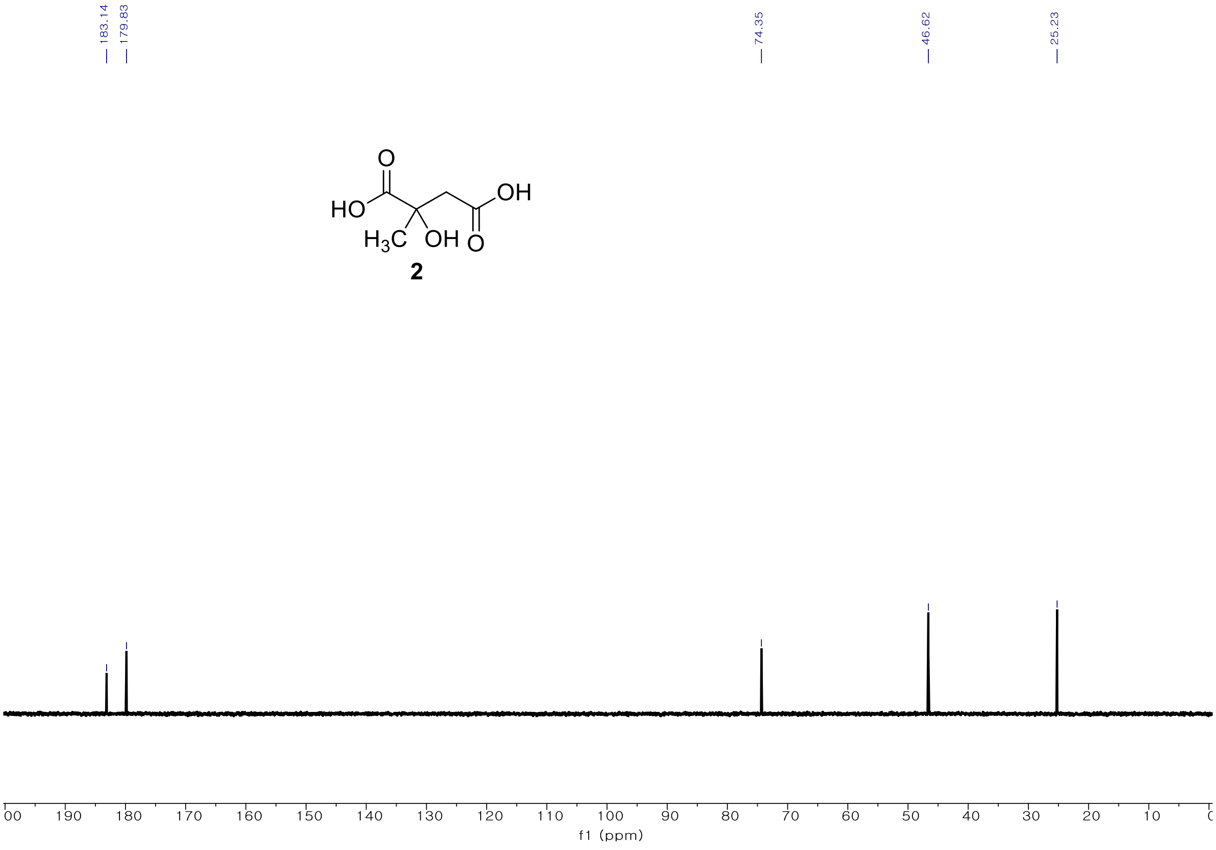

Succinic acid (3)

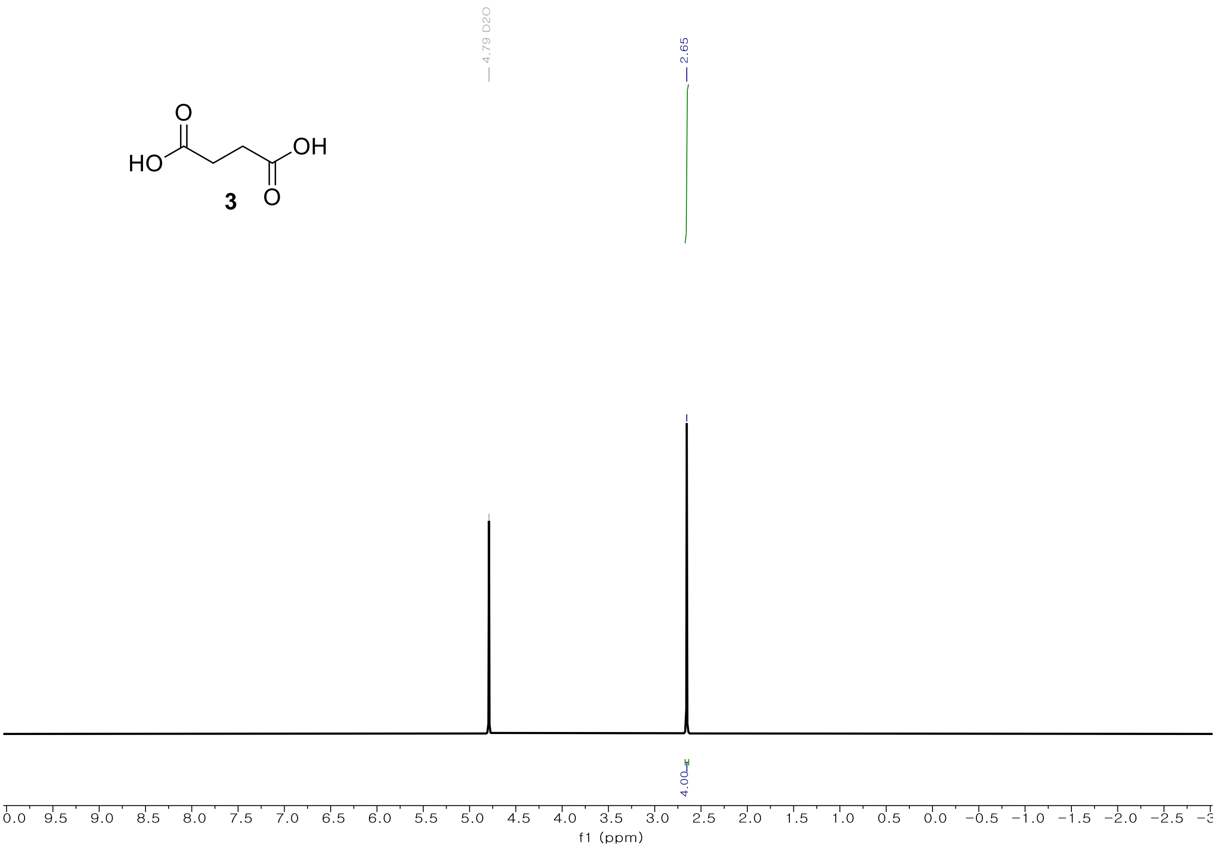

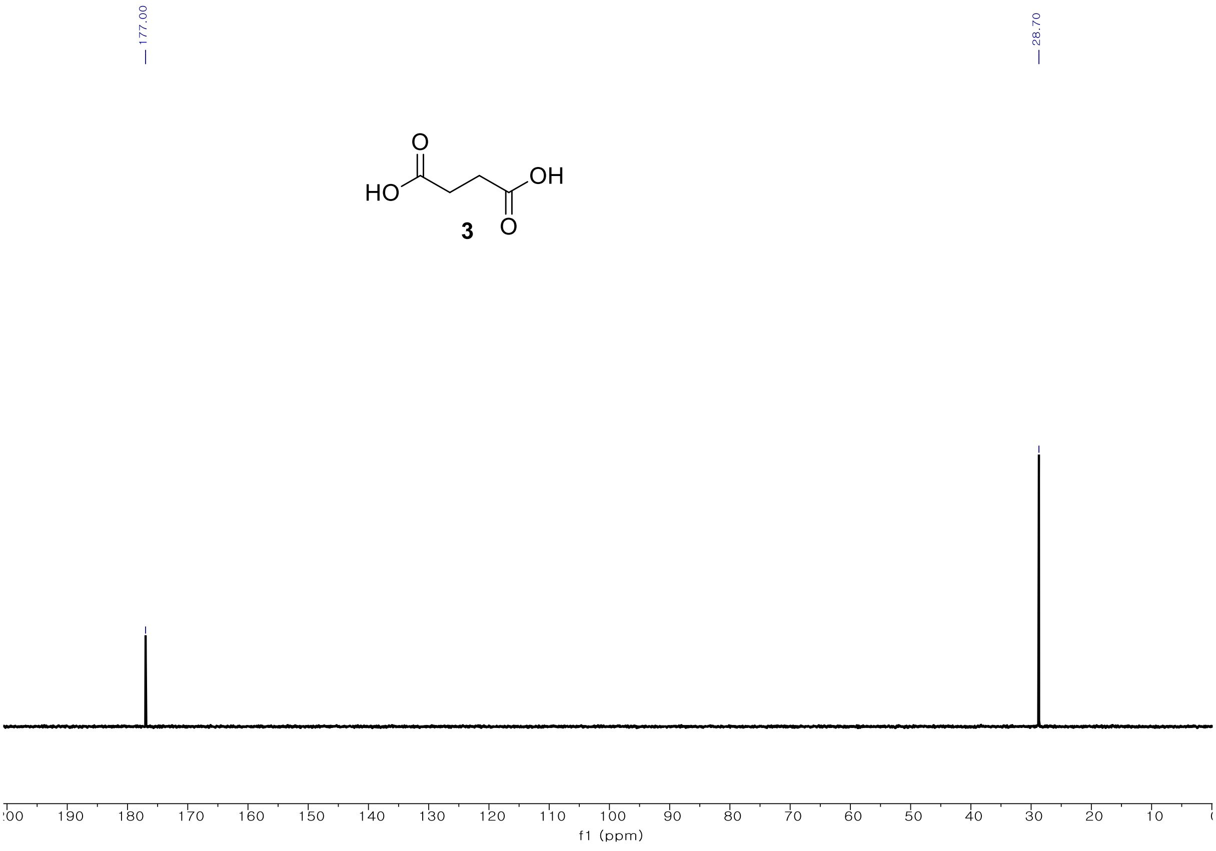

Dimethyl citramalate (20)

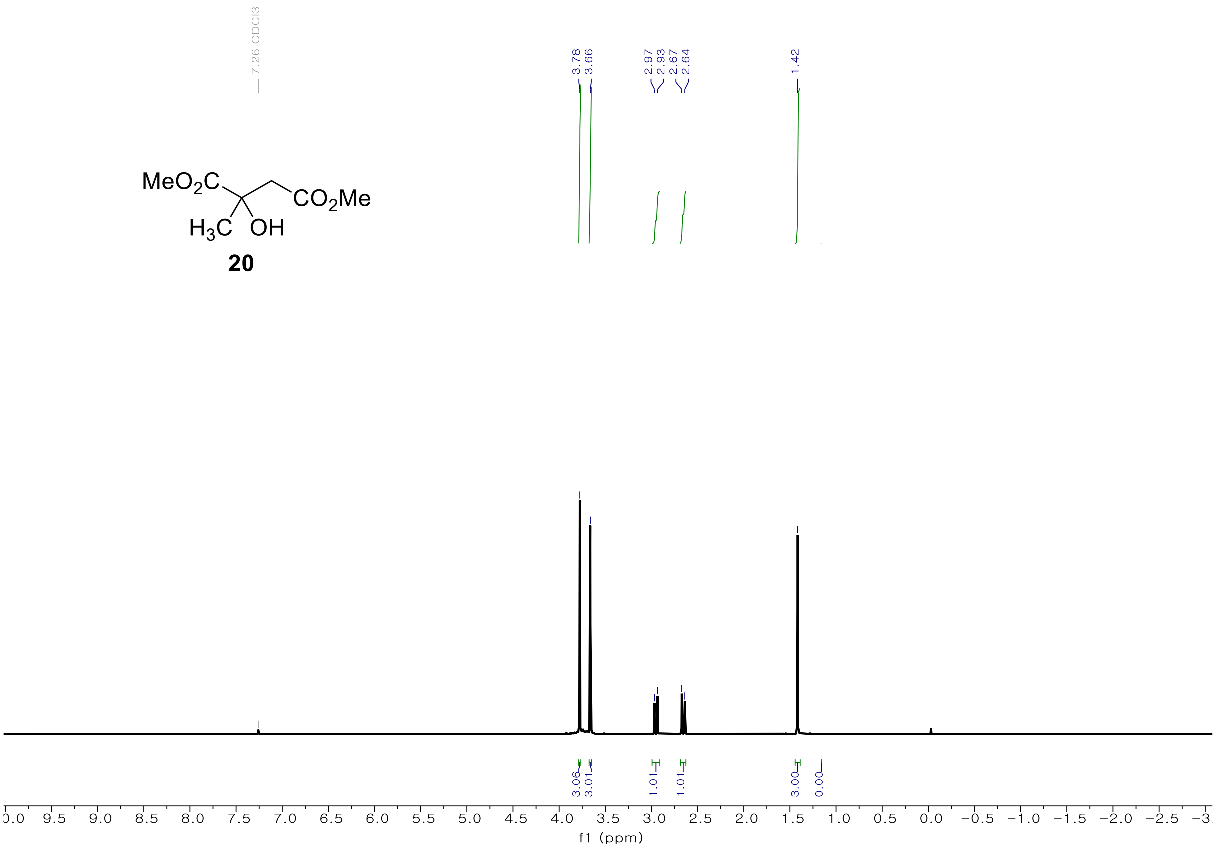

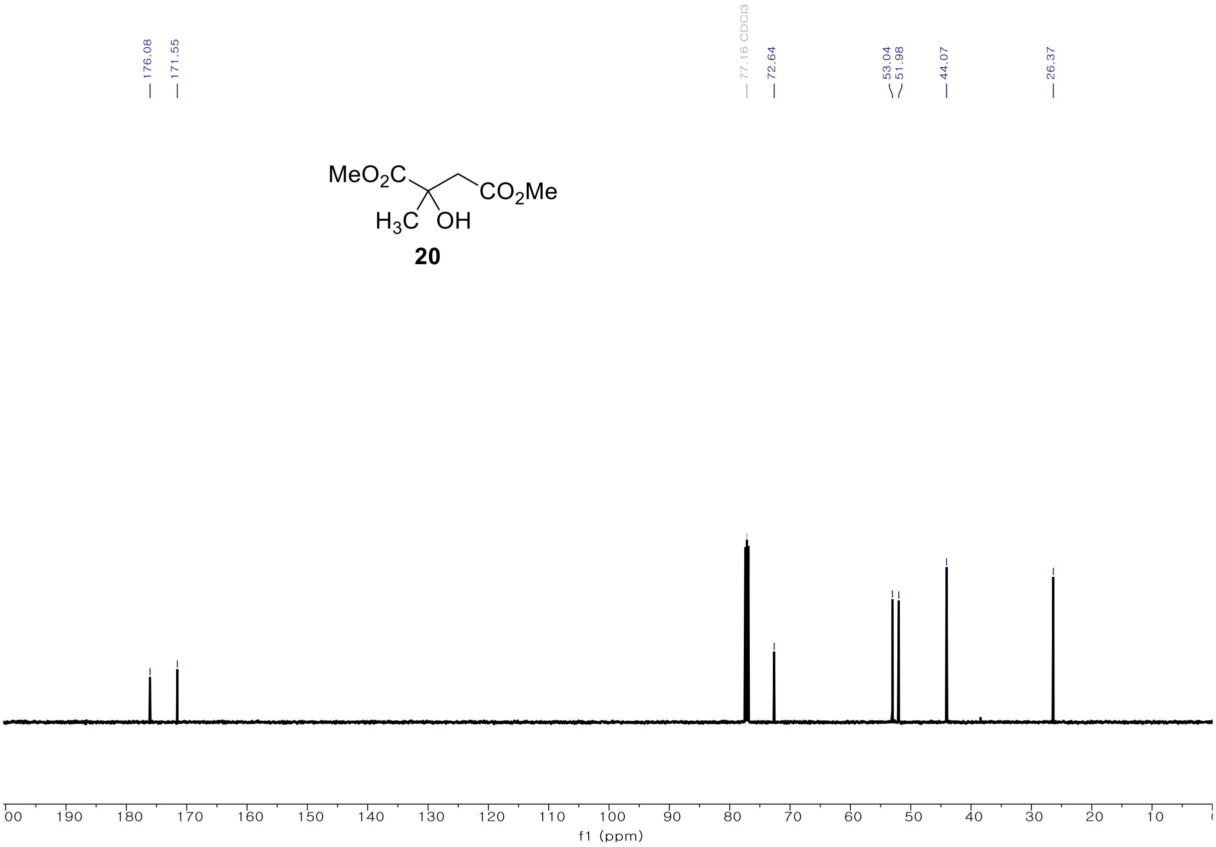

Dimethyl 2-acetamido-2-methylsuccinate (21)

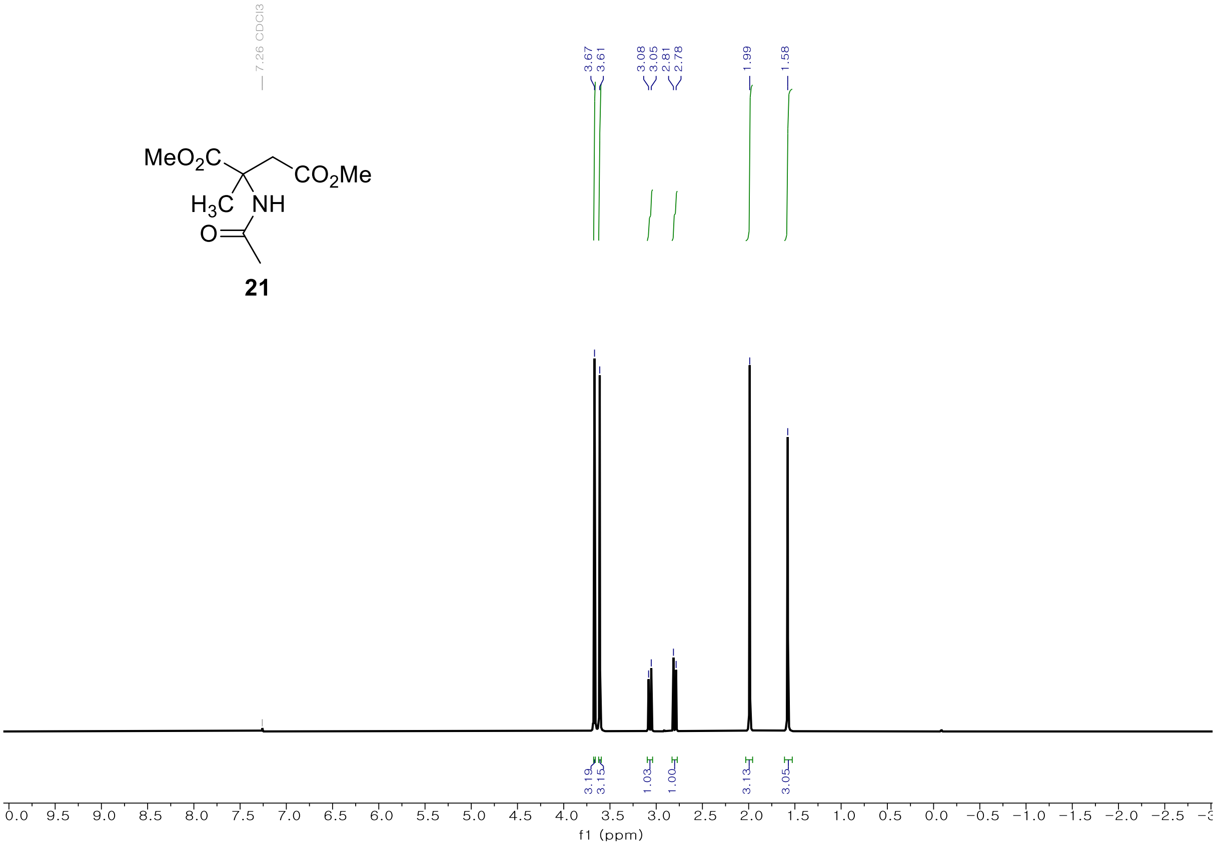

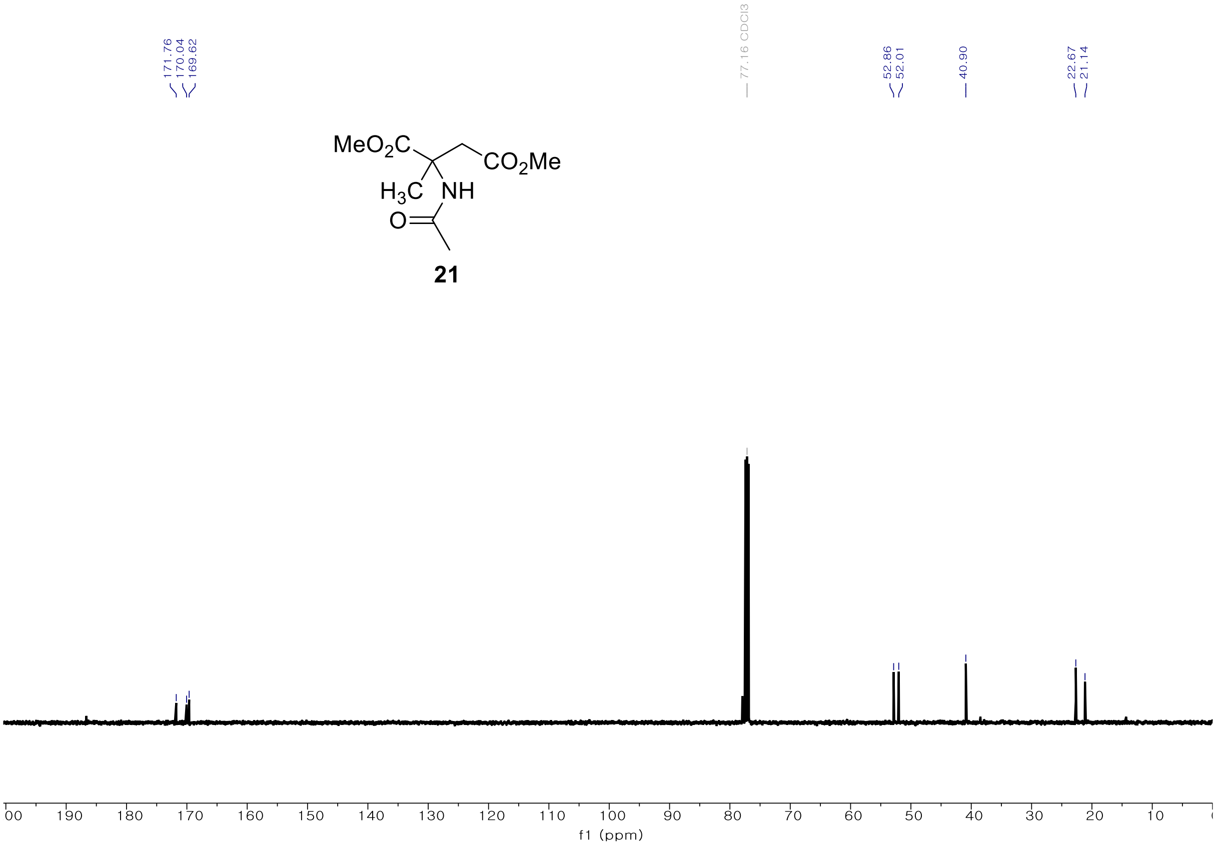

Dimethyl 2-amino-2-methylsuccinate (22)

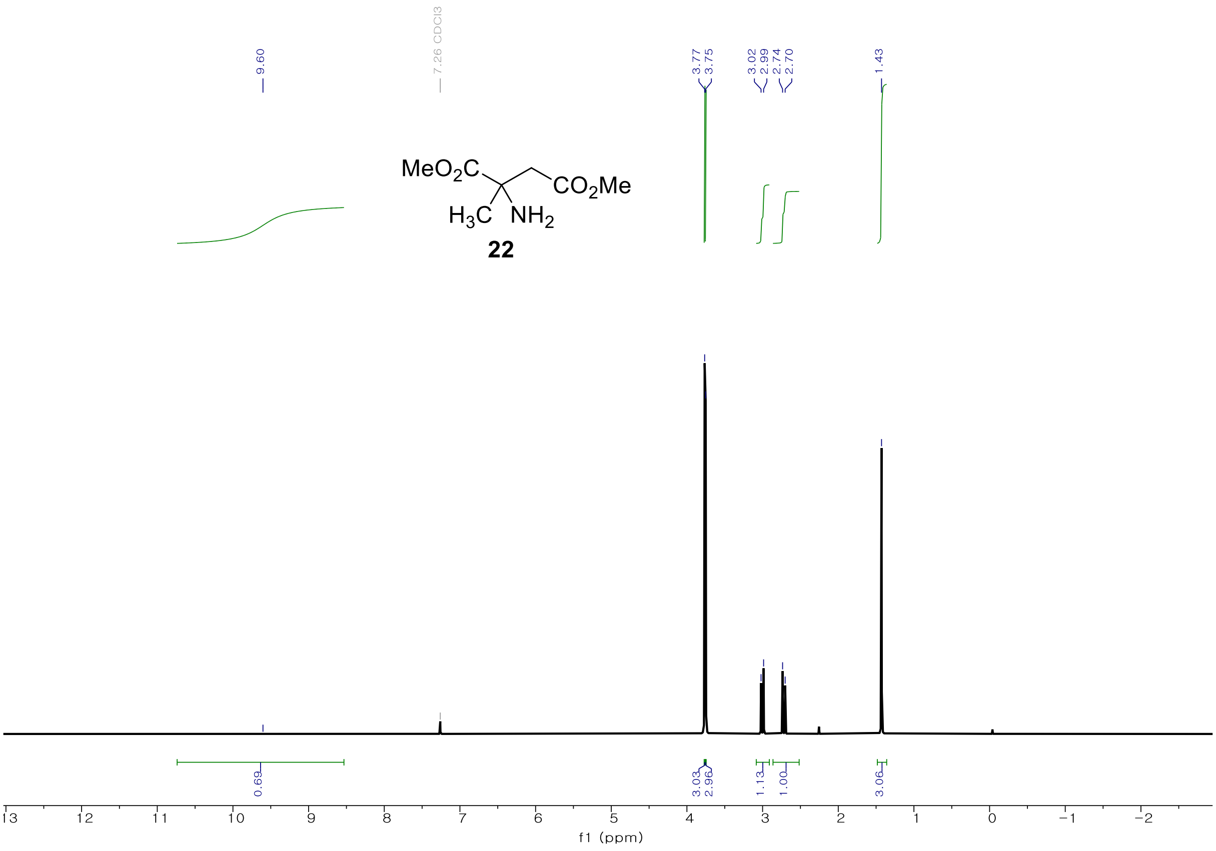

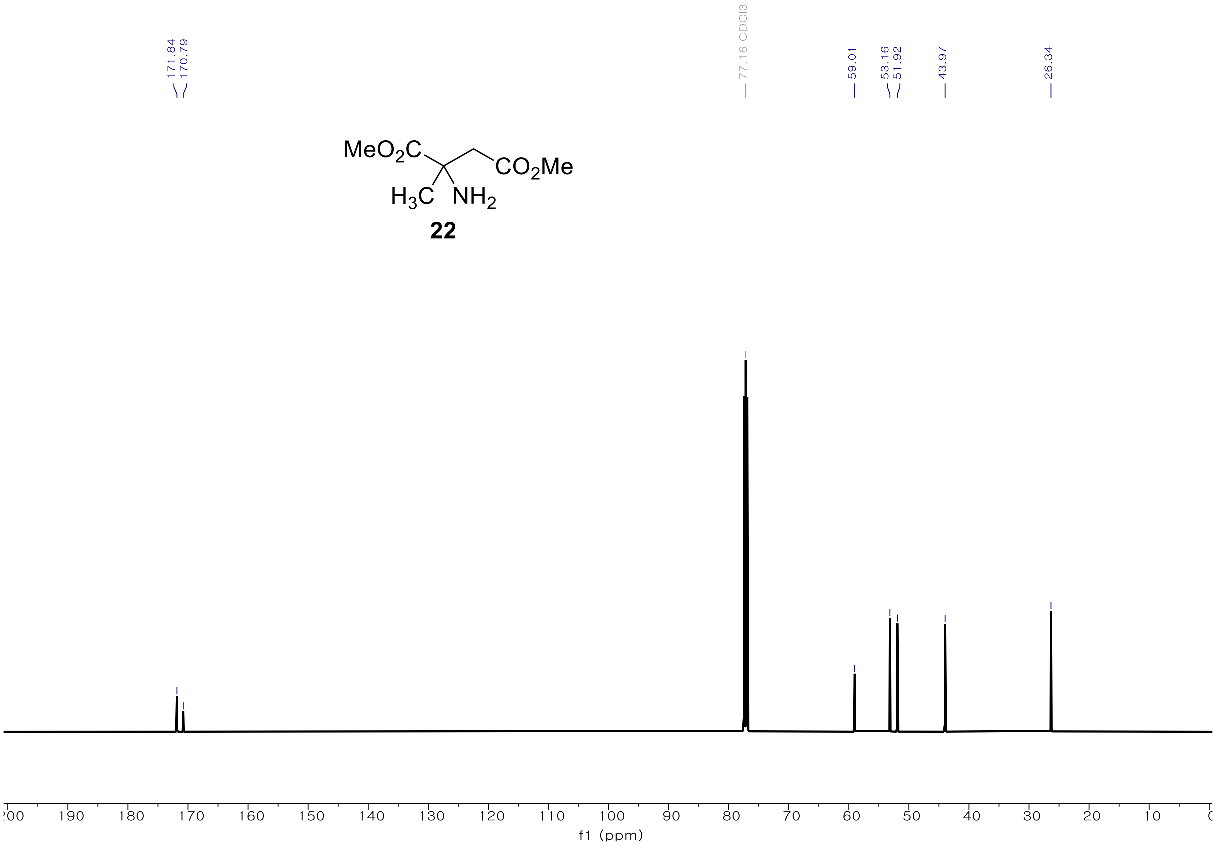

Itaconic acid (23)

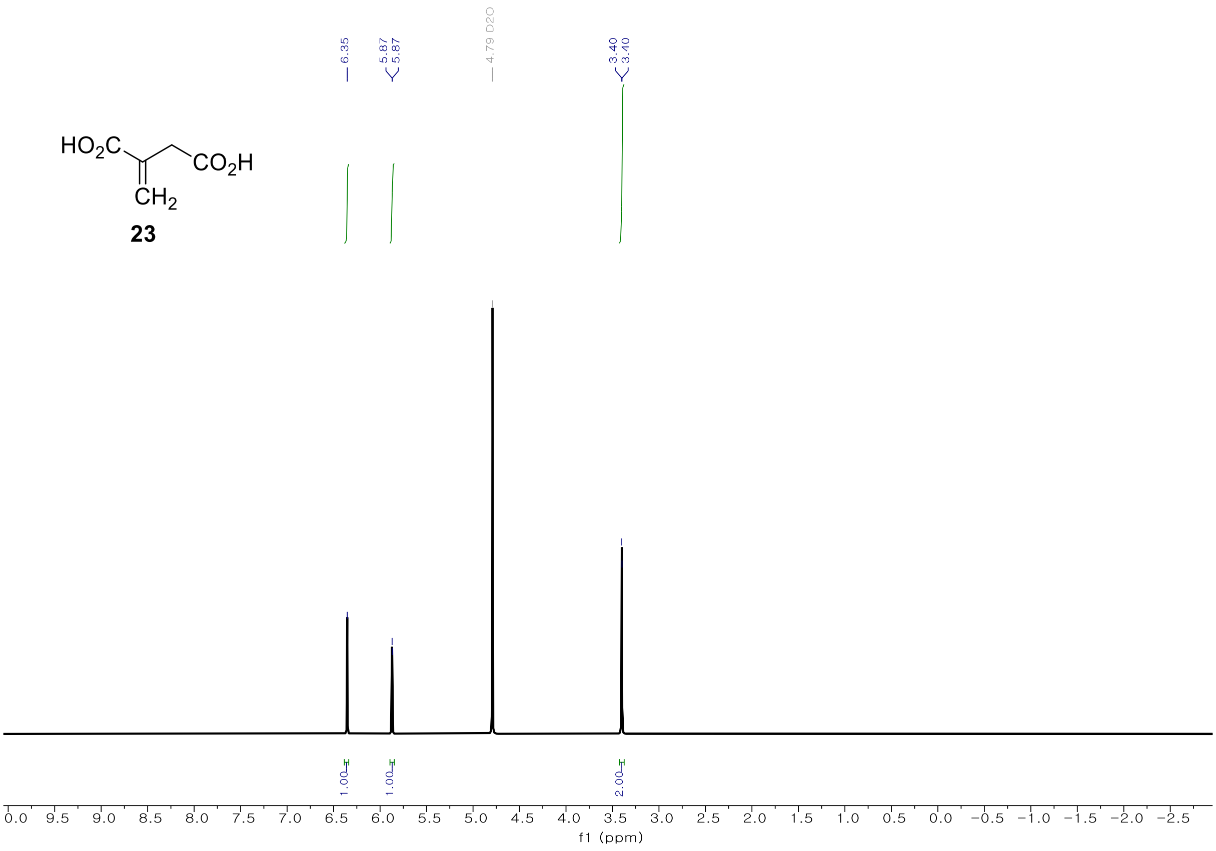

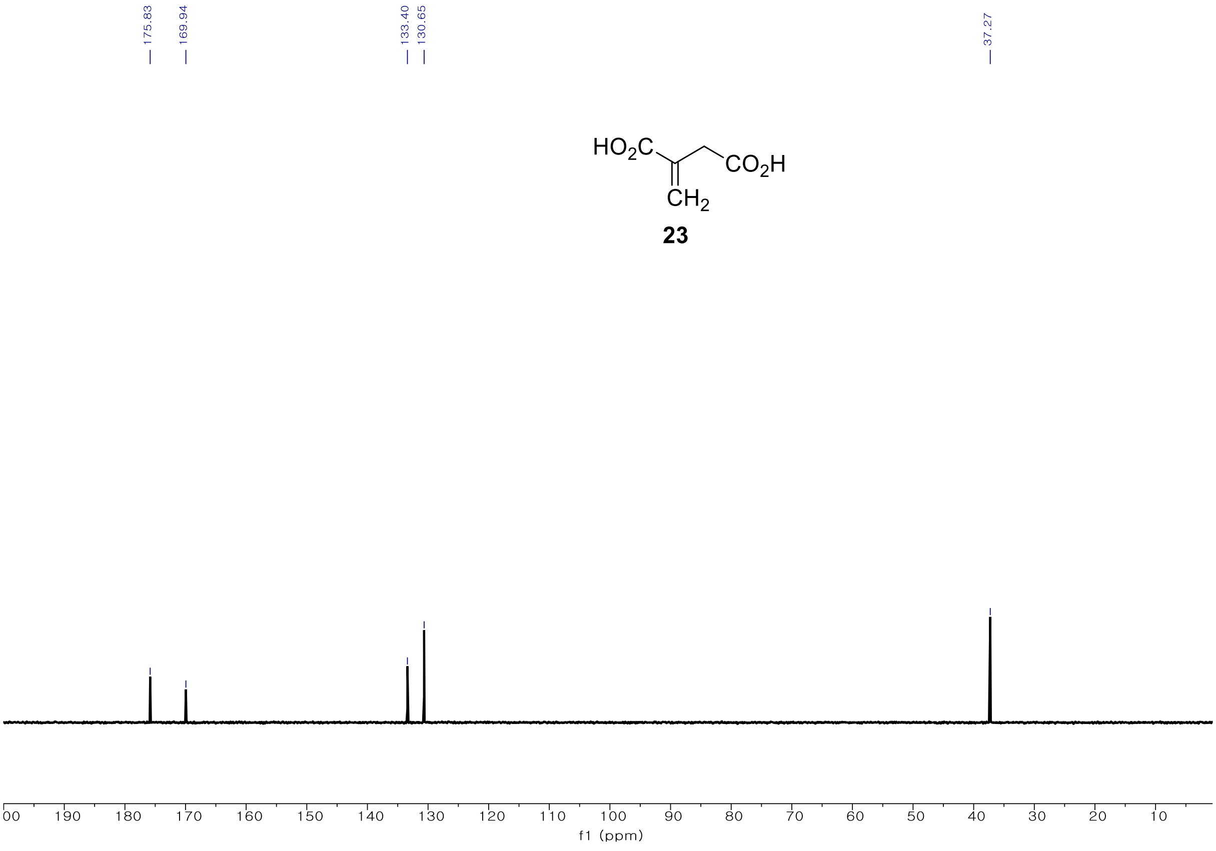

Acetic acid (A)

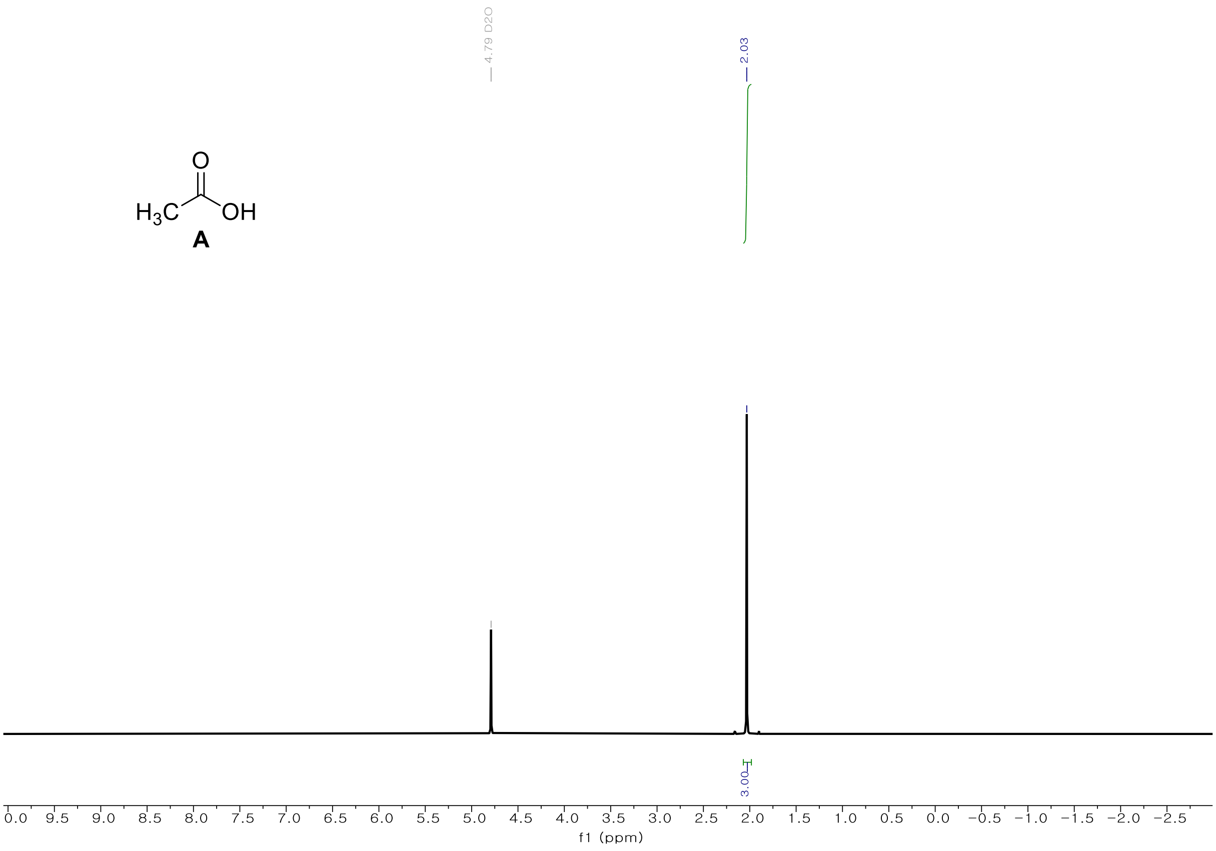

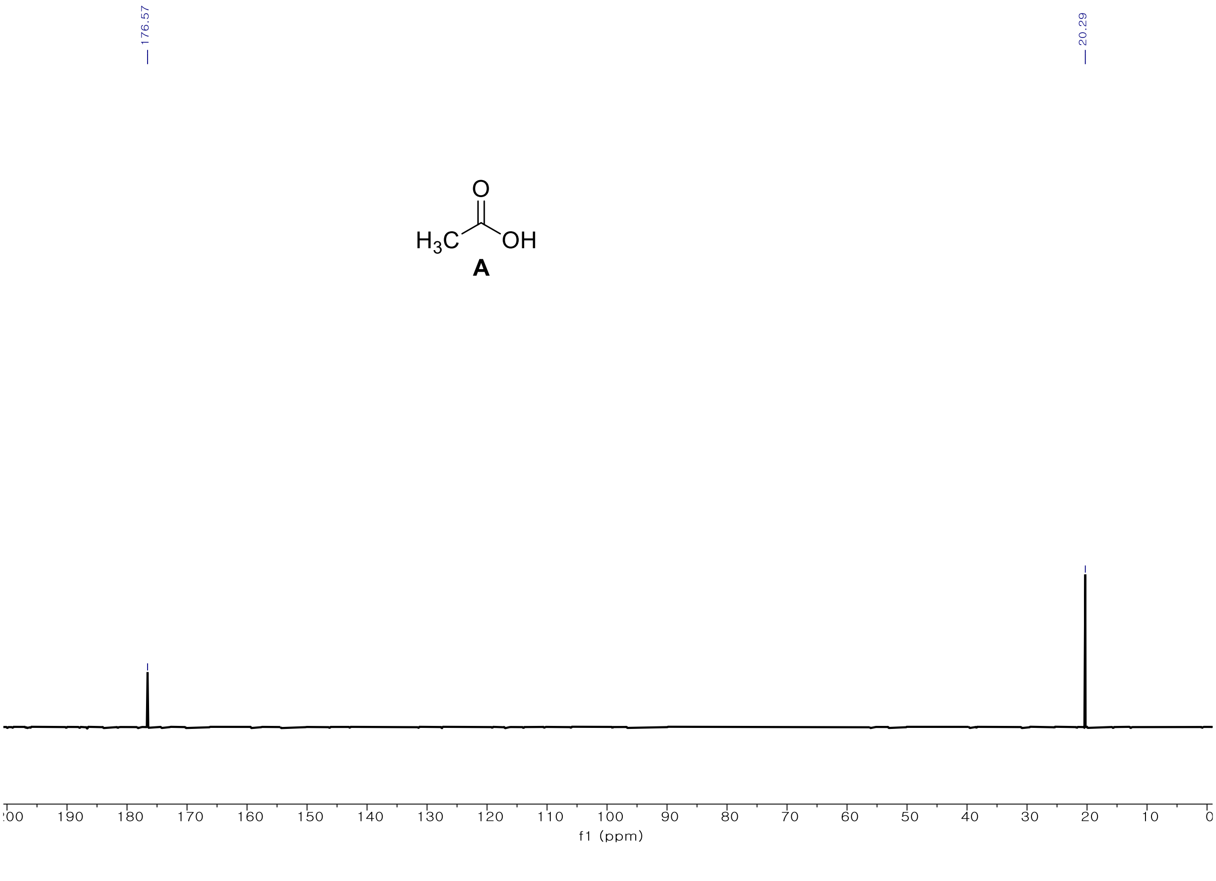

Formic acid (B)

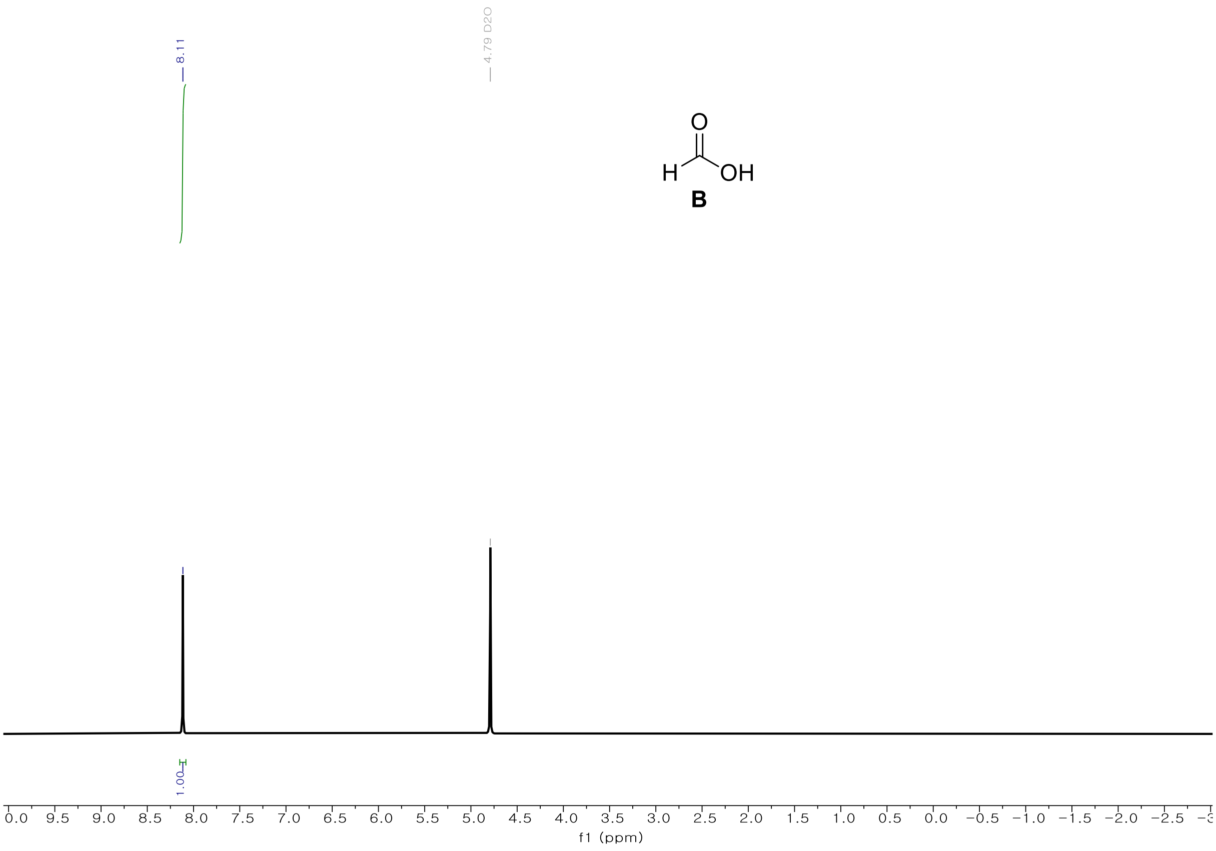

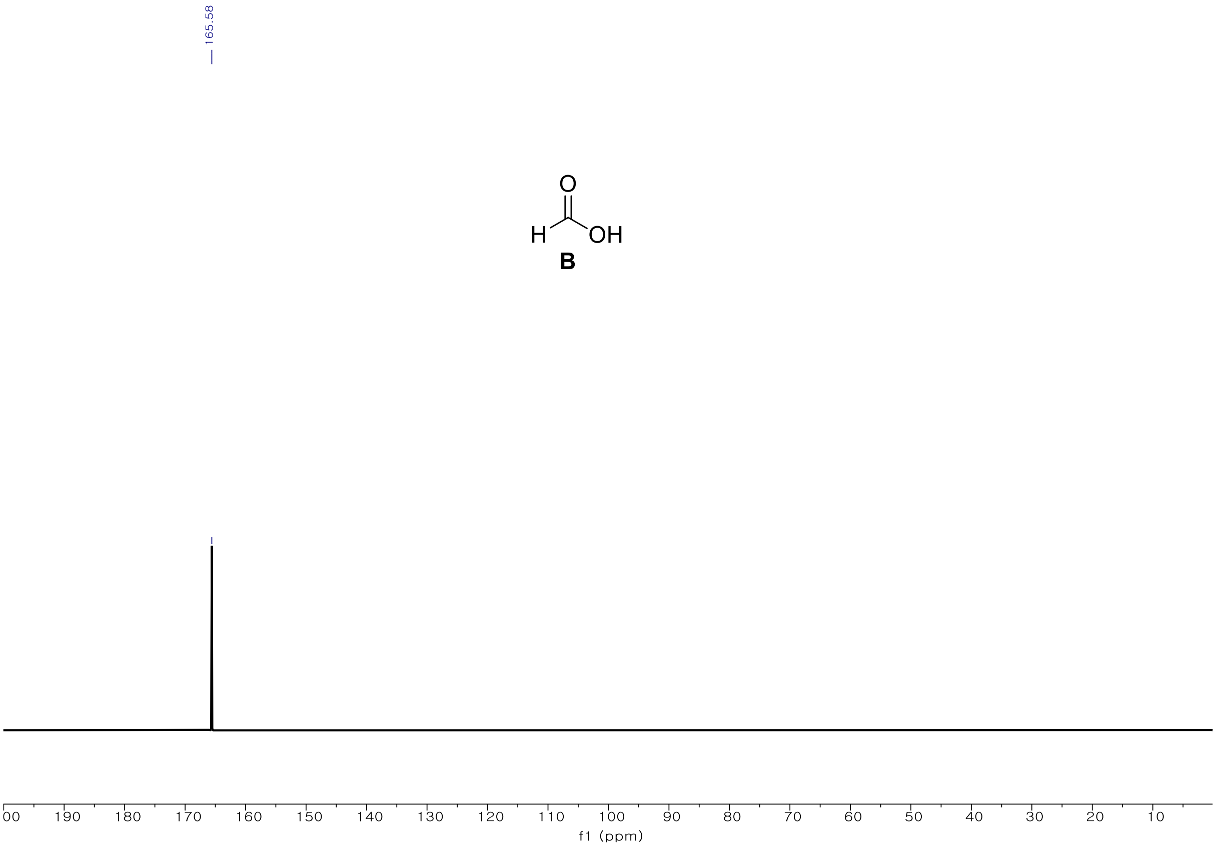

Salicylic acid (D)

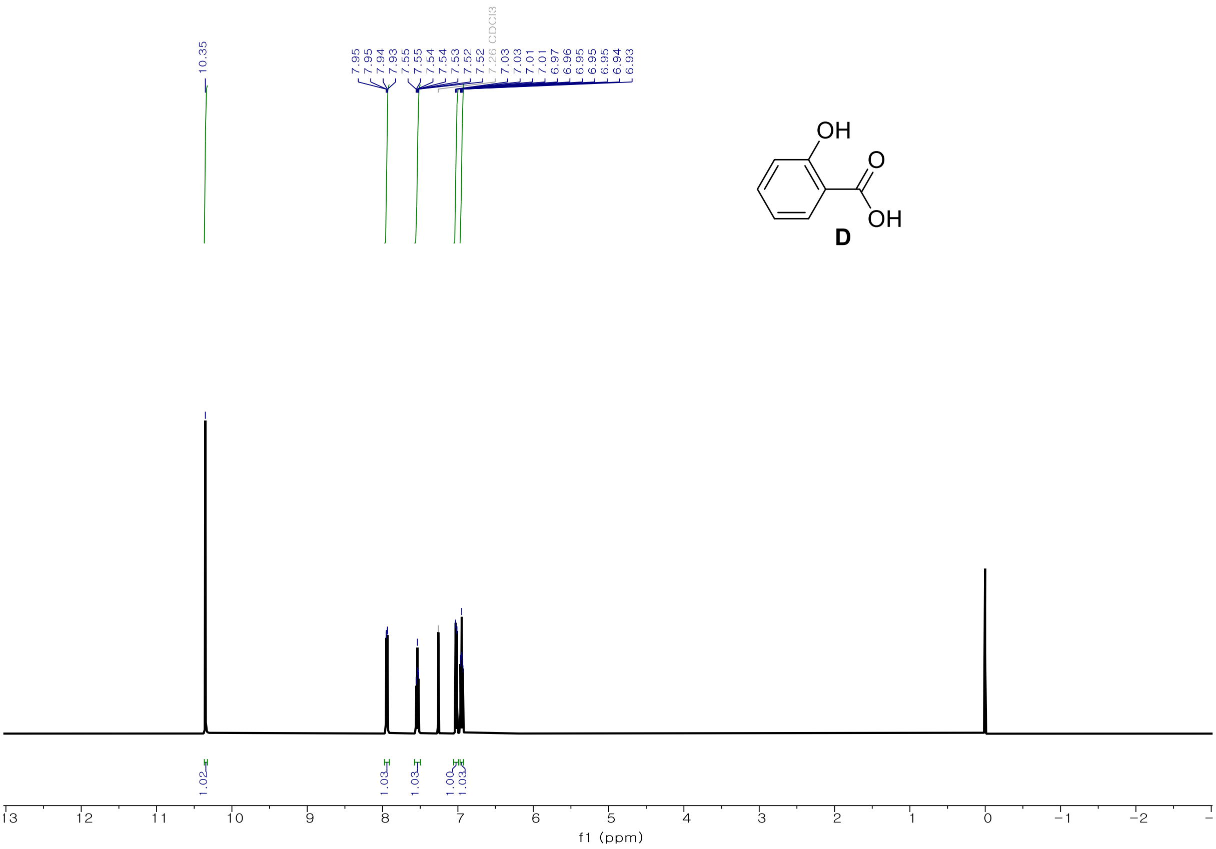

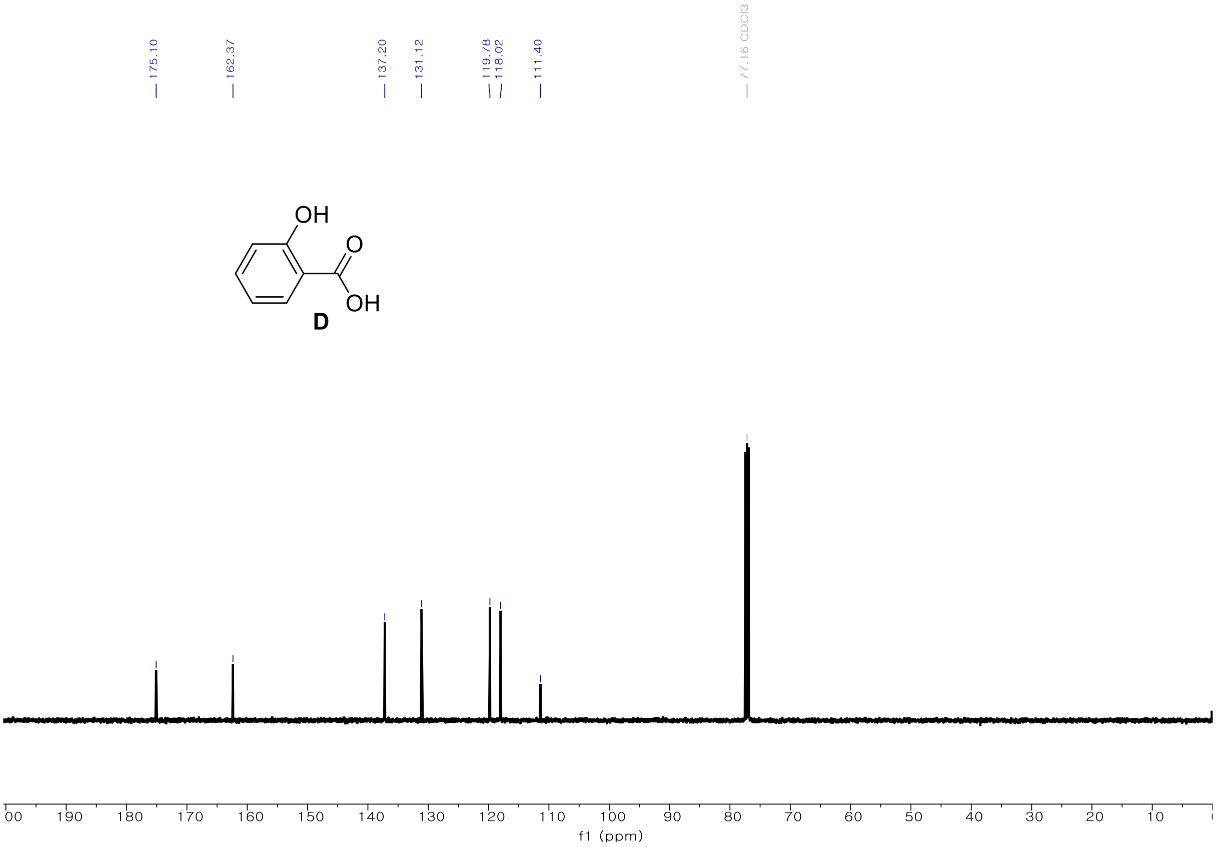

2,2,6,6-Tetramethylpiperidin-1-ol [TEMPO-H (E)]

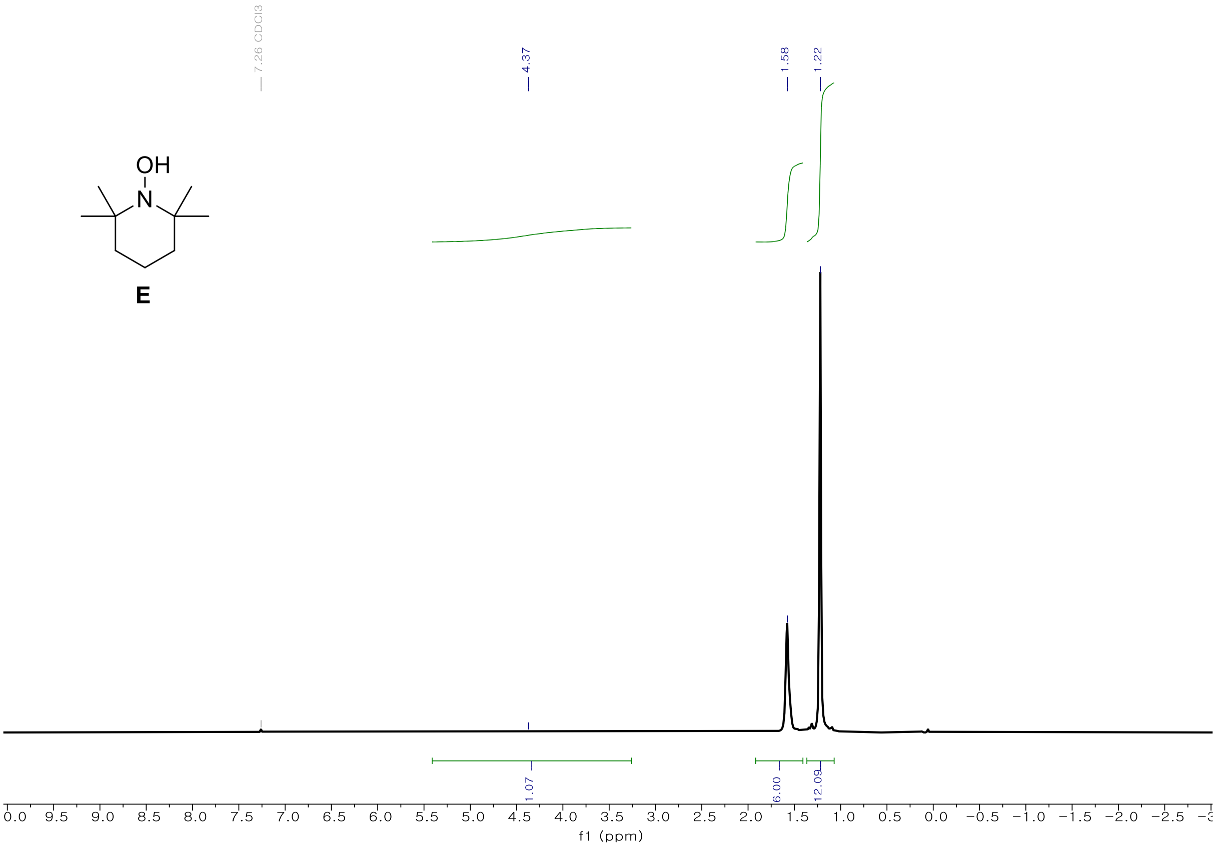

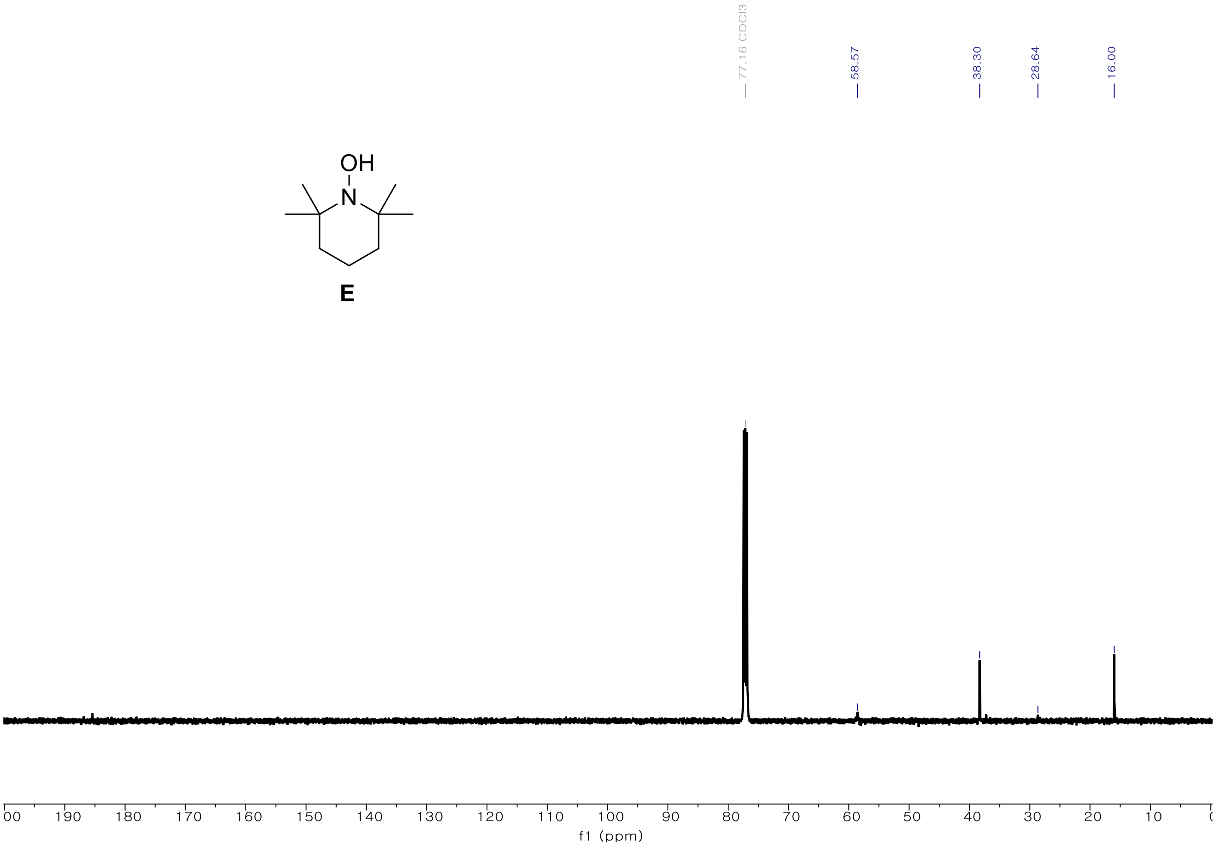

2-Hydroxy-2-phenylsuccinic acid (G)

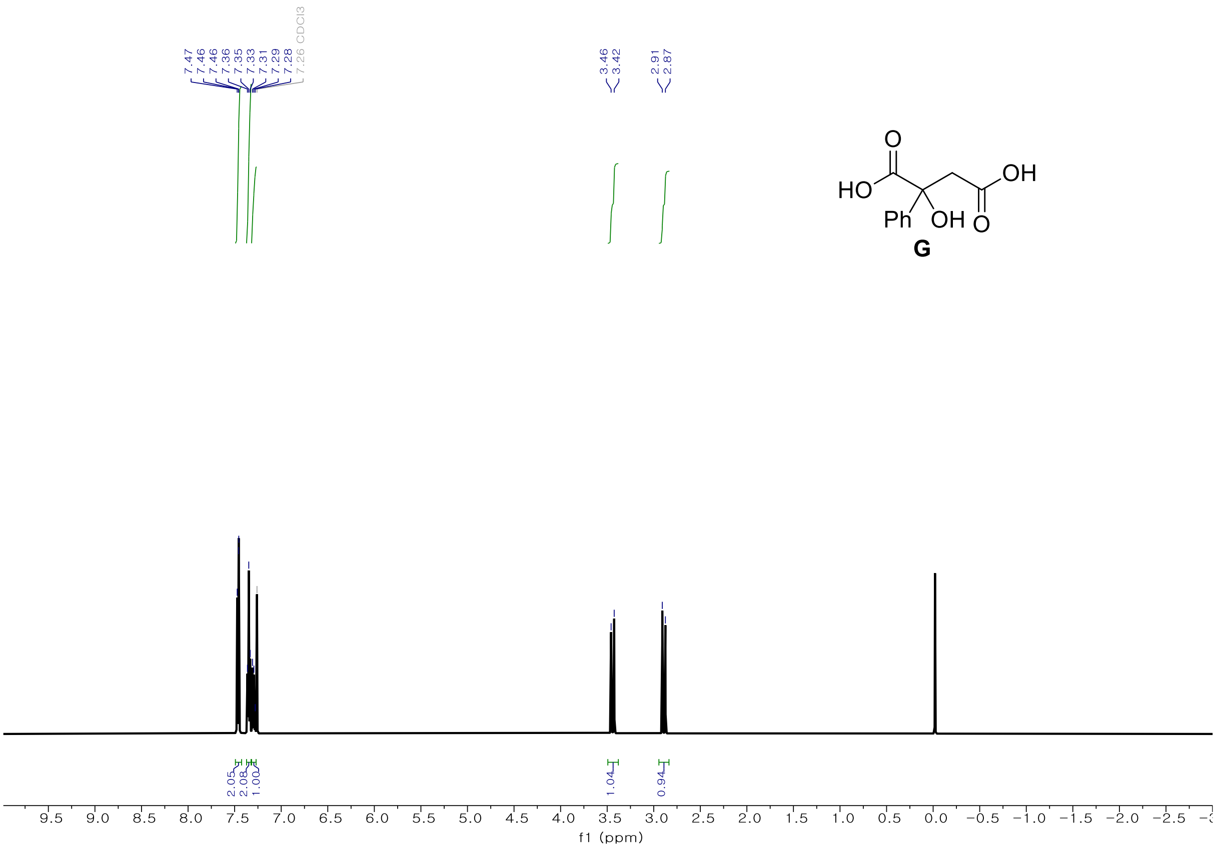

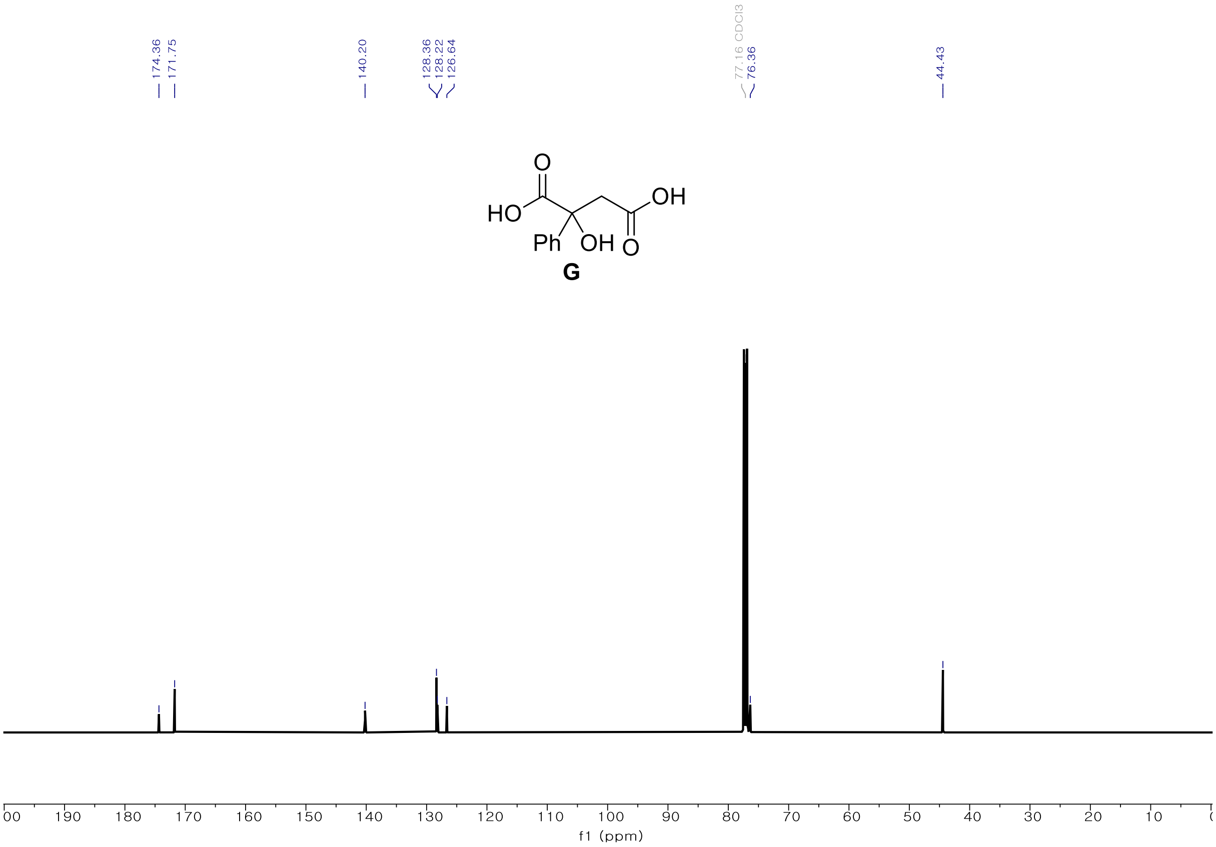

Supplement: Supplementary file 1 — Supplementary Material [file CSSC-19-e202502757-s001.pdf]
